# Distribution of GABAergic Neurons and VGluT1 and VGAT Immunoreactive Boutons in the Ferret (*Mustela putorius*) Piriform Cortex and Endopiriform Nucleus. Comparison With Visual Areas 17, 18 and 19

**DOI:** 10.3389/fnana.2019.00054

**Published:** 2019-05-31

**Authors:** Daniela Navarro, Mayvi Alvarado, Alejandra Figueroa, Cristina Gonzalez-Liencres, Federico Salas-Lucia, Pablo Pacheco, Maria V. Sanchez-Vives, Pere Berbel

**Affiliations:** ^1^Departamento de Histología y Anatomía, Facultad de Medicina, Universidad Miguel Hernández (UMH), Alicante, Spain; ^2^Instituto de Neuroetología, Universidad Veracruzana, Xalapa, Mexico; ^3^Instituto de Neurociencias, UMH-Consejo Superior de Investigaciones Científicas (CSIC), Alicante, Spain; ^4^Àrea Neurociència de Sistemes, Institut d’Investigacions Biomèdiques August Pi i Sunyer (IDIBAPS), Barcelona, Spain; ^5^Institució Catalana de Recerca i Estudis Avançats (ICREA), Generalitat de Catalunya, Barcelona, Spain

**Keywords:** NeuN, calcium-binding proteins, epilepsy, piriform network, visual cortex

## Abstract

We studied the cellular organization of the piriform network [comprising the piriform cortex (PC) and endopiriform nucleus (EP)] of the ferret (*Mustela putorius*)—a highly excitable region prone to seizures—and, more specifically, the distribution and morphology of different types of gamma-aminobutyric acid (GABA)ergic neurons, and the distribution and ratio of glutamatergic and GABAergic boutons, and we compared our findings to those in primary visual area 17, and secondary areas 18 and 19. We accomplished this by using cytochrome oxidase and immunohistochemistry for mature neuronal nuclei (NeuN), GABAergic neurons [glutamic acid decarboxylase-67 (GAD67), calretinin (CR) and parvalbumin (PV)], and for excitatory (vesicular glutamate transporter 1; VGluT1) and inhibitory (vesicular GABA transporter; VGAT) boutons. In the ferret, the cellular organization of the piriform network is similar to that described in other species such as cats, rats and opossums although some differences also exist. GABAergic immunolabeling showed similarities between cortical layers I–III of the PC and visual areas, such as the relative distribution of GABAergic neurons and the density and area of VGluT1- and VGAT-immunoreactive boutons. However, multiple differences between the piriform network and visual areas (layers I–VI) were found, such as the percentage of GABAergic neurons with respect to the total number of neurons and the ratio of VGluT1- and VGAT-immunoreactive boutons. These findings are relevant to better understand the high excitability of the piriform network.

## Introduction

Ferrets (*Mustela putorius*) are a useful model for research on cortical development and plasticity because they are born with an immature state of corticogenesis compared to other mammals such as monkeys, cats and rodents (Jackson et al., 1989; Chapman et al., 1996; Noctor et al., 1997). Although anatomical studies characterizing the cerebral cortex in adult ferrets are relatively scarce, some reports have described the anatomical and functional organization in several primary and associative sensory cortical areas (Weliky et al., 1995; McLaughlin et al., 1998; Innocenti et al., 2002; Manger et al., 2002a,b, 2004; Dell et al., 2019a,b,c). These studies have uncovered some functional similarities with other mammals such as rats (Ekstrand et al., 2001b), opossums (Haberly, 1983), cats (Witter et al., 1988) and monkeys (Neville and Haberly, 2004).

For instance, adult ferrets, cats and monkeys show a similar tangential distribution of callosal projections. Callosal neurons are located in the border between visual areas 17 and 18, and in disperse regions in areas 18 and 19 (Essen and Zeki, 1978; Innocenti et al., 2002). Recent studies described also similarities in cortical (ipsi- and contra-lateral) and thalamic connectivity between the occipital (Dell et al., 2019a), temporal (Dell et al., 2019b) and postero-parietal (Dell et al., 2019c) visual cortical areas in the ferret, using standard anatomical tract-tracing methods. However, some differences have been also reported, such as the lack of the striae of Baillarger characteristic of other mammals including cats and monkeys, and the fewer callosal projecting neurons in the border between visual areas 17 and 18 compared to cats (Otsuka and Hassler, 1962; Essen and Zeki, 1978; Innocenti et al., 2002). Despite the interest for developmental and functional studies, data on the gamma-aminobutyric acid (GABA)ergic and excitatory/inhibitory organization of the cortex in adult ferrets are scarce.

Particularly interesting because of the excitatory/inhibitory organization are the piriform cortex (PC) and the adjacent endopiriform nucleus, which are reciprocally connected (O’Leary, 1937; Valverde, 1965; Luskin and Price, 1983a; Behan and Haberly, 1999), and comprise what is often considered one functional unit: the piriform network (Sanchez-Vives et al., 2008). In the ferret, the piriform network is located in the rostral ventral region of the brain and is delimited dorsally by the rhinal sulcus. The PC is a three-layered olfactory sensory area (Ramón y Cajal, 1911; O’Leary, 1937; Valverde, 1965; Stevens, 1969; Haberly, 1983, 1990), also involved in associative and behavioral responses (Neville and Haberly, 2004). In vertebrates, layer I of the PC contains very few cells and consists of layer Ia, which receives afferents from the olfactory bulb, and layer Ib, which receives afferents from the anterior olfactory nucleus and the entorhinal cortex (Powell et al., 1965; White, 1965; Luskin and Price, 1983a; Manger et al., 2002a; Haberly, 1990). Layer II contains densely packed cells and receives afferents from olfactory areas, the dorsal peduncular cortex, the ventral *tenia tecta* and the periamygdaloid complex. In layer III are sparsely distributed cells that receive afferents from the same areas as layer Ib (Luskin and Price, 1983b). Underneath layer III is the endopiriform nucleus containing more densely packed cells than layer III, and medial to the endopirifom nucleus is the claustrum (Wang et al., 2017). In addition to connections with olfactory areas, the piriform network is reciprocally connected with the entorhinal cortex, the amygdaloid nuclei, the mediodorsal and medial thalamic nuclei and the nucleus accumbens (Behan and Haberly, 1999; Kowiański et al., 1999b; Vismer et al., 2015).

A remarkable feature of the PC is its proneness to generating epileptic discharges (Hoffman and Haberly, 1993, 1996) because of its high excitability (Piredda and Gale, 1985). In fact, the *area tempestas* in the posterior PC is able to generate seizures when chemically or electrically stimulated (Löscher and Ebert, 1996). In agreement with this, electrophysiological studies in the ferret have found faster speed of neural activity propagation and higher excitability in the PC (Sanchez-Vives et al., 2008) than in primary visual area 17 and secondary visual areas 18 and 19 (Sanchez-Vives and McCormick, 2000; Massimini et al., 2004; Capone et al., 2019). The increased horizontal speed of propagation could be due to the contribution of different factors such as the axon caliber (Innocenti and Caminiti, 2017), the existence of long-distance running collaterals allowing the transfer of neural activity without neuron-to-neuron breaks (Johnson et al., 2000), or less negative values of GABA_A_ reversal potentials providing less inhibition in the PC (Kapur et al., 1997). The higher excitability, in addition, is likely directly related to the excitatory/inhibitory balance, whereby GABAergic inhibitory interneurons play a critical role (Compte et al., 2003; Sanchez-Vives et al., 2008, 2010). However, the reasons behind the proneness to seizures, the faster propagation speed and higher excitability in the piriform network than in the neocortex are not yet clear, and the probable role of the inhibitory system has not yet been uncovered.

To gain insights into the cytoarchitectonic and functional organization of the inhibitory system in the ferret PC and endopiriform nucleus, and its involvement in epilepsy, excitability and propagation of neural information in mammalian seizures, we studied the morphology and distribution of two types of GABAergic neurons such as parvalbumin (PV) and calretinin (CR) immunoreactive (ir) neurons, as well as the distribution and ratio of vesicular glutamate transporter 1 (VGluT1)-ir (excitatory inputs) and vesicular GABA transporter (VGAT)-ir boutons (inhibitory inputs) in the PC and endopiriform nucleus, and we compared these data with our findings in visual areas 17, 18 and 19. Our results provide a better understanding of the excitatory/inhibitory system in the piriform network.

## Materials and Methods

### Ethics Statement

Animal care and drug administration were performed under veterinary control and in accordance with the European Union guidelines on protection of vertebrates used for experimentation (Directive 2010/63/EU of the European Parliament and of the Council of 22 September 2010) and all experiments were approved by the Ethics Committee at the Universitat de Barcelona.

### Animals, Tissue Processing and Immunohistochemistry

Thirteen ferrets (*Mustela putorius*; 2–12 months old, either sex) were included in this study. Ferrets were housed in a temperature-controlled (22–24°C) animal care facility, with 12-h cycles of light and darkness, and with food and water *ad libitum*.

After being deeply anesthetized with sodium pentobarbital (40 mg/kg) and inhaling 1.5%–2% isoflurane (Laboratorios Dr Esteve, S.A., Barcelona, Spain) in O_2_ (0.9 L/min), animals were intracardially perfused with 50 mL saline followed by 200 mL 4% paraformaldehyde, 0.02% CaCl_2_ and 0.1 M sucrose in 0.1 M phosphate buffer (pH 7.3–7.4). The brains were removed and post-fixed by immersion in phosphate buffer at room temperature for 4 h and stored at 4°C in 0.05% sodium azide in phosphate buffer (PB-azide).

**Figure 1:**
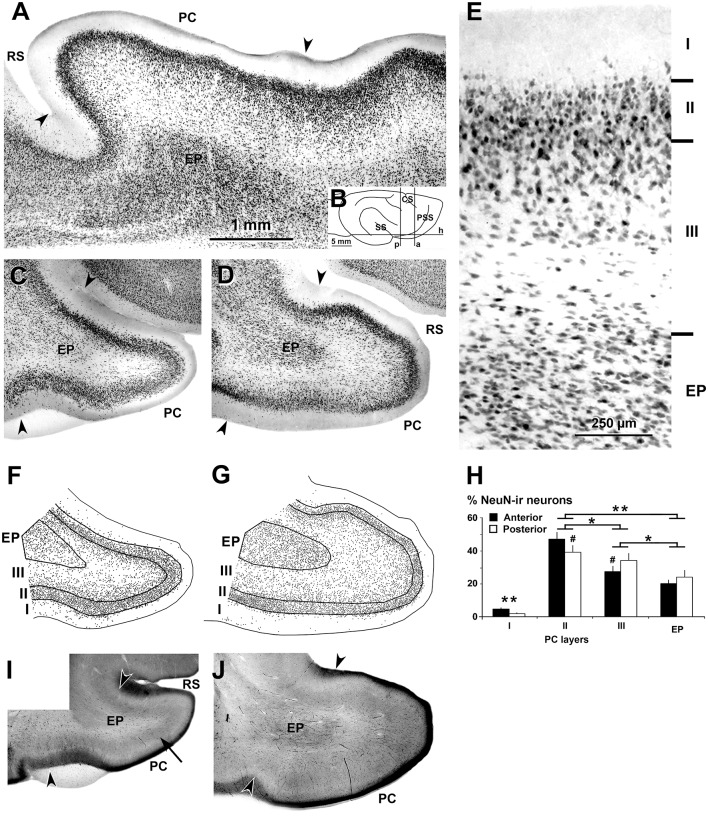
Neuronal nuclei (NeuN) immunostaining and cytochrome oxidase histochemistry in the piriform network. Photomicrographs of horizontal **(A)** and coronal **(C–E)** sections show NeuN immunostaining in the piriform cortex (PC) and endopiriform nucleus (EP). **(B)** Cartoon shows the coronal anterior (a), coronal posterior (p) and horizontal (h) planes of cutting. Anterior **(C,F)** and posterior **(D,E,G)** sections are shown. **(A,C,D)** Low magnification photomicrographs show a high density of neurons in PC layers II and upper III, and in EP. **(E)** High magnification photomicrograph shows small densely packed neurons in PC layer II and EP. **(F,G)** Plots show the distribution of NeuN-ir neurons. **(H)** Bar chart shows the percentages of NeuN-ir neurons in PC and EP. A higher percentage of NeuN-ir neurons in PC layer II with respect to other PC layers and EP can be seen. The percentage of neurons was higher in posterior PC layer II and lower in layer III than in the anterior counterparts. **(I,J)** Cytochrome oxidase staining shows a heavy cytochrome oxidase-stained band in layers II and upper III in the PC (arrow in **I**). Borders between layers **(E,F,G)** and limits with adjacent areas (arrowheads in **A,C,D,I,J**) are indicated. RS, Rhinal sulcus; CS, Cruciate sulcus; SS, Sylvian sulcus; PSS, Pre-sylvian sulcus. Same magnification for **(A,C,D,F,G,I,J)**. Vertical lines in bar charts show SD. ^#^,*P < 0.05, **P < 0.001.

For the piriform studies, one hemisphere of each brain was cut coronally from anterior to posterior in a vibratome at 100 μm, except for three animals in which it was cut horizontally from ventral to dorsal at 100 μm. To increase the number of samples and assess possible significant differences between anterior and posterior regions of the PC and endopiriform nucleus, two sets of coronal section were taken. One set was taken at the coronal plane located by the end of the crucial sulcus and the second set, 3.2–4 mm more posteriorly (Figure 1B cited out of order here). For visual studies, the other hemisphere was cut parasagittally from medial to lateral at 50 μm (Figure 8A cited out of order here). In each case, eight parallel series of sections were obtained and stored in PB-azide at 4°C.

**Figure 2:**
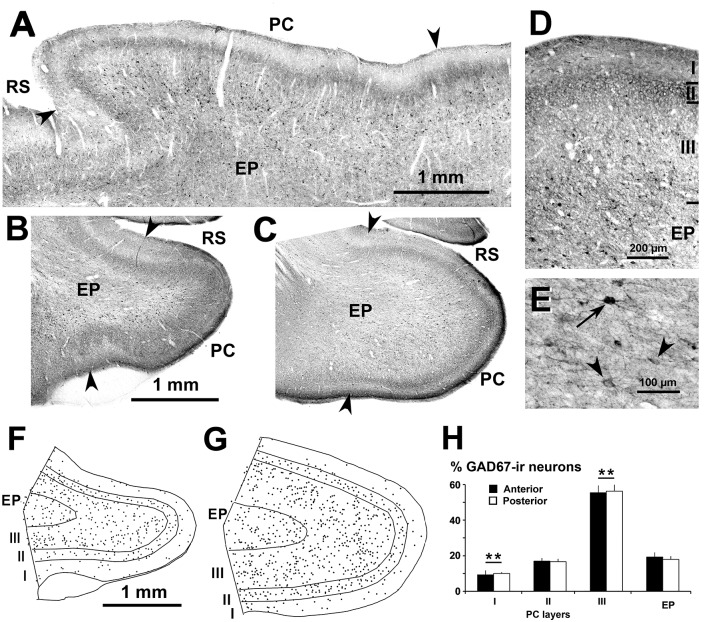
Distribution of glutamic acid decarboxylase-67 (GAD67)-ir neurons in the piriform network. Photomicrographs of horizontal **(A)** and coronal **(B–E)** sections show GAD67-ir neurons in the PC and endopiriform nucleus (EP). Anterior **(B)** and posterior **(C–E)** sections are shown. **(D)** At higher magnification, many processes and perisomatic boutons in layer II can be observed. **(E)** Heavily (arrow) and lightly (arrowhead) stained GAD67-ir neurons in EP can be seen. **(F,G)** Plots show the distribution of GAD67-ir neurons. **(H)** Bar chart shows that the percentage of GAD67-ir neurons in PC significantly decreased from layer III to layer I. No other significant differences between layer II and EP, and between anterior and posterior sections were found. Borders between layers **(D,F,G)** and limits between adjacent areas (arrowheads in **A–C**) are indicated. Same magnification for **(B,C)** and **(F,G)**. RS, Rhinal sulcus. Vertical lines in bar charts show SD. **P < 0.001.

**Figure 3:**
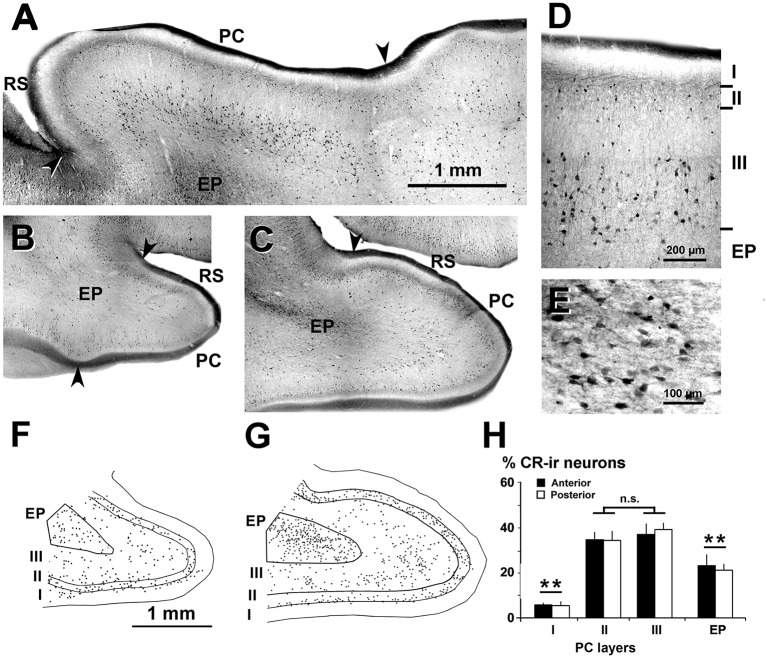
Distribution of CR-ir neurons in the piriform network. Photomicrographs of horizontal **(A)** and coronal **(B–E)** sections show CR-ir neurons in the PC and endopiriform nucleus (EP). Anterior **(B)** and posterior **(C–E)** sections are shown. **(D,E)** At higher magnification, many processes and boutons in layers I and II can be seen. **(D)** Most CR-ir neurons in layer II resemble bipolar cells, while in layer III, they are small and large multipolar. **(E)** In EP, CR-ir neurons are multipolar and fusiform bipolar, and in the neuropil, numerous perisomatic boutons can be observed. **(F,G)** Plots show the distribution of CR-ir neurons. **(H)** Bar chart shows a high percentage of CR-ir neurons in PC layers II and III with respect to other layers and EP. No significant differences were found between the anterior and posterior sections. Borders between layers **(D,F,G)** and limits with adjacent areas (arrowheads in **A–C**) are indicated. RS, Rhinal sulcus. Same magnification for **(B,C,F,G)**. Vertical lines in bar charts show SD. n.s, not significant differences. **P < 0.001.

**Figure 4:**
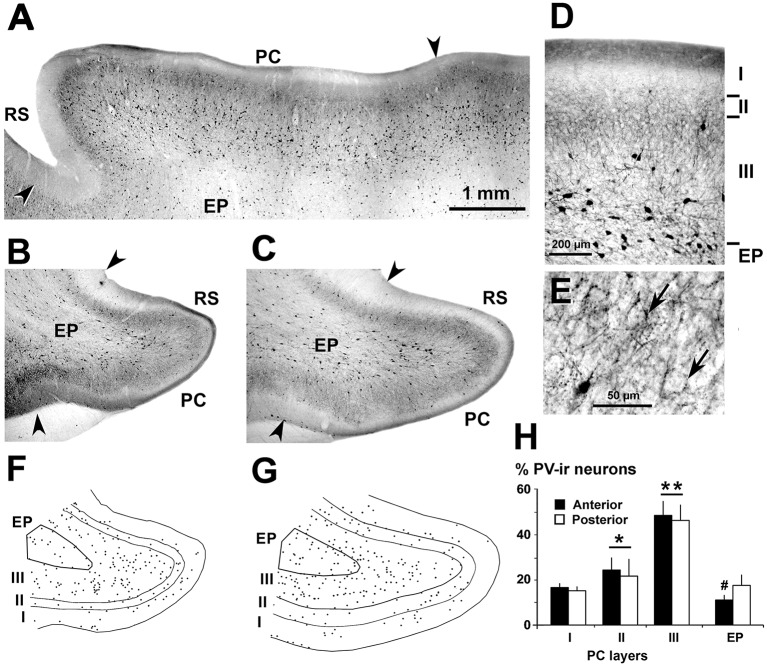
Distribution of parvalbumin (PV)-ir neurons in the piriform network. Photomicrographs of horizontal **(A)** and coronal **(B–E)** sections show CR-ir neurons in the PC and endopiriform nucleus (EP). Anterior **(B,F)** and posterior **(C–E,G)** sections are shown. **(D,E)** At higher magnification, many processes and boutons in PC layers II and III can be seen. **(D)** Most PV-ir neurons in layer II resemble bipolar with vertically oriented processes, while in layer III they resemble small and large multipolar. In EP, small multipolar PV-ir neurons can be seen. **(E)** In PC layers II and upper III, and in EP, numerous terminal-like perisomatic boutons can be seen. **(F,G)** Plots show the distribution of PV-ir neurons. **(H)** Bar chart shows a higher percentage of PV-ir neurons (anterior and posterior sections averaged) in layers II and III with respect to layer I and EP. The PV-ir neuron percentage was higher in the posterior than in the anterior EP and was similar in layer I and EP. Borders between layers **(D,F,G)** and limits with adjacent areas (arrowheads in **A–C**) are indicated. RS, Rhinal sulcus. Same magnification for **(A,B,C,F,G)**. Vertical lines in bar charts show SD. ^#^,*P < 0.05, **P < 0.001.

**Figure 5:**
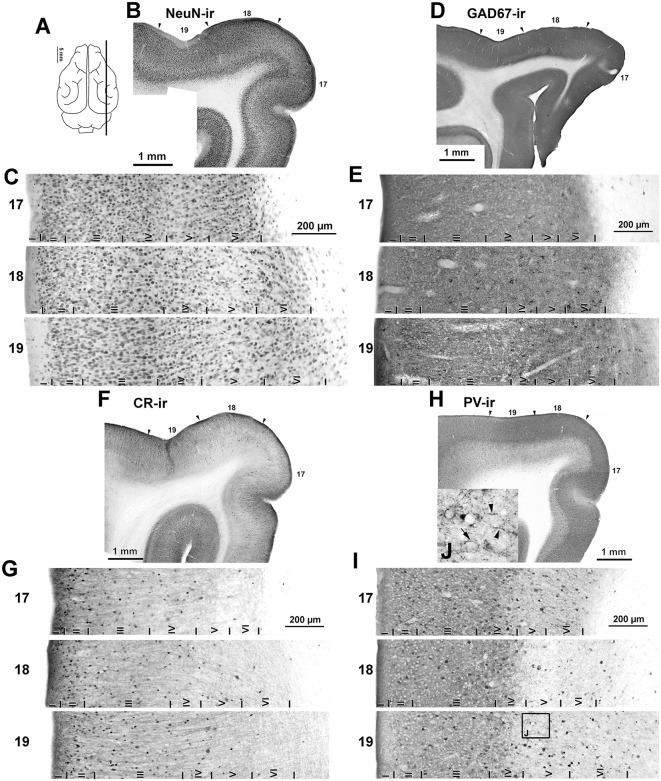
Immunostaining of NeuN-ir, GAD67-ir, CR-ir and PV-ir neurons in visual areas 17, 18 and 19. **(A)** The schematic upper view of the ferret cerebral hemispheres shows the parasagittal plane of cutting. **(B,D,F,H)** Low-power photomontages show the borders (arrowheads) between visual areas 17, 18 and 19. **(C,E,G,I)** Photomicrographs show detail of the distribution of NeuN-ir, GAD67-ir, CR-ir and PV-ir neurons in areas 17, 18 and 19. Borders between layers I through VI (vertical lines) are indicated. **(C)** Note the clear border between layers of visual areas in NeuN immunostained sections. **(H,I)** Note the increased PV immunostaining in layers I–IV. **(J)** High magnification (inset of layer V in area 19) shows numerous perisomatic (arrow) and chandelier-like (arrowheads) boutons. The **(J)** box is also shown in Supplementary Figure S1.

**Figure 6:**
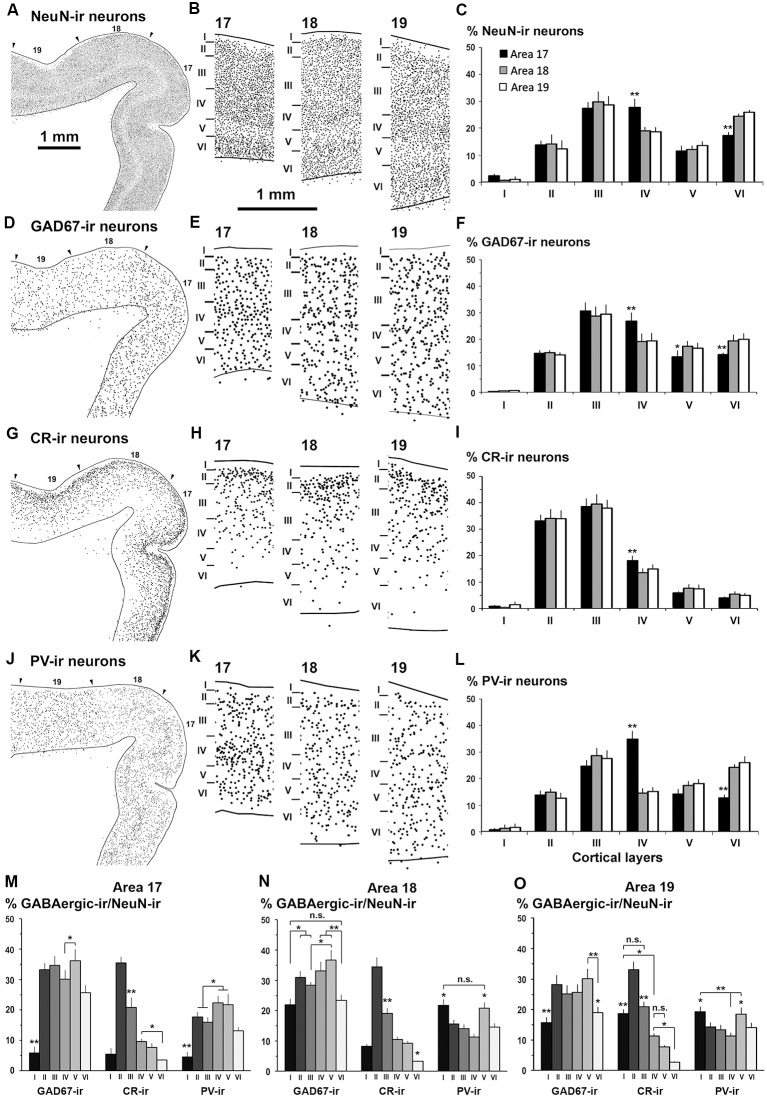
Distribution of NeuN-ir, GAD67-ir, CR-ir and PV-ir neurons in visual areas 17, 18 and 19. **(A,D,G,J)** Low-power plots show the radial distribution of NeuN-ir, GAD67-ir, CR-ir and PV-ir neurons (dots) in visual areas 17, 18 and 19 (arrowheads point the limit between visual areas). **(B,E,H,K)** Detailed plots show the radial distribution of labeled neurons. The borders between cortical layers are indicated by horizontal bars. Note the decreased density of NeuN-ir neurons in layer V and the increased density of CR-ir neurons in layer II. **(C,F,I,L)** Bar charts show the percentages of labeled neurons across cortical layers in each visual area. Comparing areas 18 and 19 with area 17, note the decrease in GAD67-, CR- and PV-ir neuron percentage in layer IV **(F,I,L)**, the increase in GAD67-ir neuron percentage in layers V and VI **(F)**, and the increase in PV-ir neuron percentage in layer VI **(L)**. The NeuN-ir neuron percentage in layer I was significantly lower than in layers II–VI. These percentages are grouped by visual area in Supplementary Figure S2. **(M,N,O)** Proportion of GAD67-ir, CR-ir and PV-ir cells in each layer with respect to NeuN-ir neurons in visual areas. Note that the distribution of proportions of CR-ir/NeuN-ir neurons was similar across areas (excluding layer I) and that the PV-ir/NeuN-ir proportion in layer IV was higher in area 17 than in areas 18 and 19. Same magnification for **(A,D,G,J)** and for **(B,E,H,K)** Vertical lines in bar charts show SD. n.s., not significant differences. *P < 0.05, **P < 0.001.

**Figure 7:**
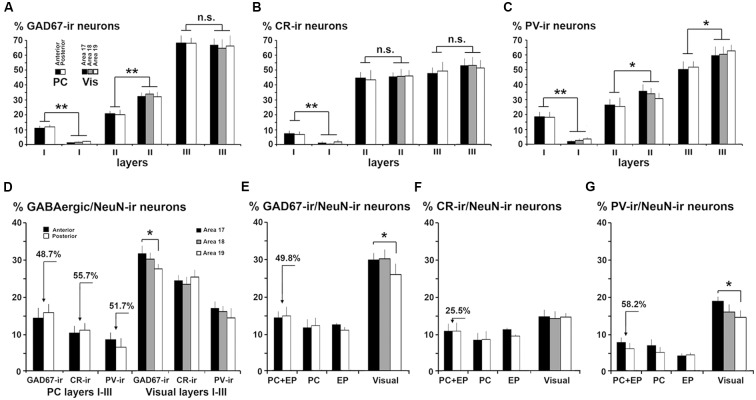
Comparison of gamma-aminobutyric acid (GABA)cergic neurons between the piriform network and visual areas 17, 18 and 19. **(A)** GAD67-ir, **(B)** CR-ir and **(C)** PV-ir neuron percentages in layers I–III in PC and visual areas 17, 18 and 19. **(B)** CR-ir neuron percentages in layers I–III were similar in PC and visual areas, although differences can be perceived in the GAD67-ir and PV-ir neuron percentages between PC layer II and visual layer II. **(D)** GAD67-ir, CR-ir and PV-ir neuron proportions in layers I–III with respect to the total number of NeuN-ir neurons decrease in PC (see arrows indicating the difference) compared to visual areas. **(E–G)** GAD67-ir, CR-ir and PV-ir neuron proportions with respect to the total number of NeuN-ir neurons in PC, endopiriform nucleus (EP) and piriform network (PC+EP) were lower than in visual areas (see arrows indicating the difference). Some differences between visual areas 17 and 18 can also be observed for GAD67-ir and PV-ir neuron percentages. Vis, visual. Vertical lines in bar charts show SD. n.s., not significant differences. *P < 0.05, **P < 0.001.

**Figure 8:**
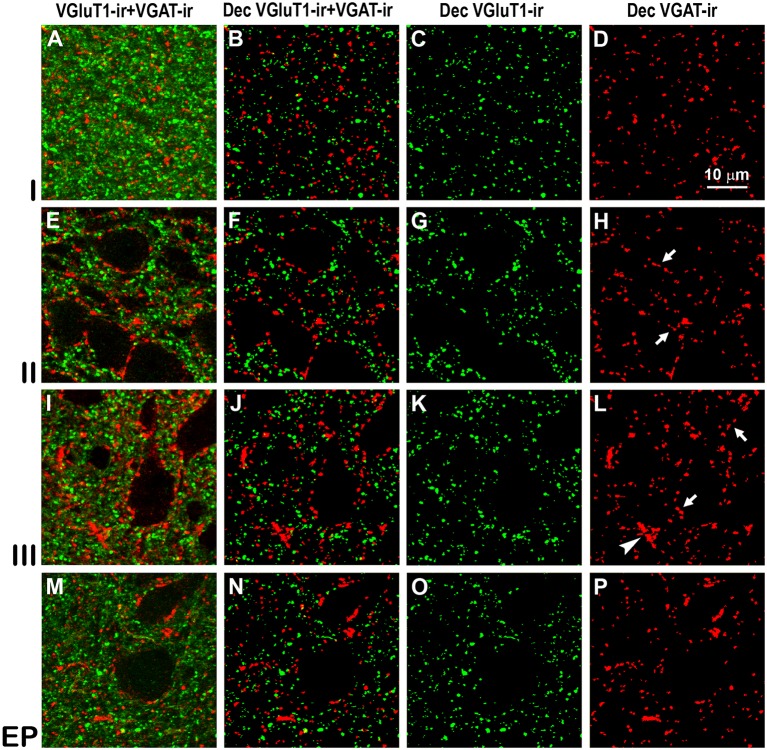
Confocal immunolabeling of vesicular glutamate transporter 1 (VGluT1)-ir and vesicular GABA transporter (VGAT)-ir boutons in the anterior piriform network. **(A–P)** Confocal photomicrographs, taken perpendicular to the pial surface, show VGluT1-ir (green) and VGAT-ir (red) boutons in the PC and endopiriform nucleus (EP). **(A,E,I,M)** Raw confocal images (VGluT1-ir+VGAT-ir). **(B,F,J,N)** Deconvoluted merged (Dec VGluT1-ir+VGAT-ir). **(C,G,K,O)** Deconvoluted VGluT1-ir (Dec VGluT1-ir). **(D,H,L,P)** Deconvoluted VGAT-ir (Dec VGAT-ir). Perisomatic (arrows) and chandelier-like (arrowhead) VGAT-ir boutons are indicated. Same scale for all images.

Series 1 and 2 were stained with 1% cresyl violet (Sigma-Aldrich Co., St. Louis, MO, USA) and for cytochrome oxidase (Wong-Riley, 1979), respectively, to delimit the borders between cortical layers and areas. Immunohistochemistry was performed in floating sections (described in detail in the Supplementary IHC protocols). Briefly, series 3 was incubated in anti-neuronal nuclei (anti-NeuN) monoclonal antibody (mAb; 1:400; Chemicon International Inc., Temecula, CA, USA), and series 4, 5 and 6 with the following markers of GABAergic neurons: anti-glutamic acid decarboxylase 67 (anti-GAD67) mAb (1:2,000; Chemicon), rabbit anti-CR polyclonal antibody (Ab; 1:2,000; Swant, Bellinzona, Switzerland) and anti-PV mAb (1:1,000; Swant), respectively. The sections were incubated with biotinylated horse anti-mouse Ab (1:150; Vector, Burlingame, CA, USA) for mAbs and with biotinylated goat anti-rabbit Ab (1:150; Vector) for Ab. The sections were then incubated with Vectastain ABC kit (1:200; both from Vector Laboratories, Inc., Burlingame, CA, USA), and 0.05% 3,3′diaminobenzidine (DAB, Sigma-Aldrich Co., St. Louis, MO, USA). The sections were mounted on gelatinized slides, air dried during 24 h, dehydrated, cleared and cover-slipped. Series 7 was double immunostained for fluorescence with guinea pig anti-VGluT1 Ab (1:5,000; Merck-Millipore, Darmstadt, Germany) and with rabbit anti-VGAT Ab (1:2,000; Synaptic Systems, Göttingen, Germany) to detect excitatory glutamatergic and inhibitory GABAergic boutons, respectively. The sections were then incubated with goat anti-guinea pig antibody, Alexa Fluor 488 labeled (1:200, Molecular Probes, Eugene, OR, USA), followed by goat biotinylated anti-rabbit antibody (1:200, Vector) and NeutrAvidin, Rhodamine Red conjugate (1 μg/mL, Molecular Probes). Twenty-four hours before examining the sections, they were mounted using ProLong Gold (Molecular Probes), studied in a Leica confocal laser fluorescence microscope and processed using the LCS Lite software. Series 8 was unprocessed.

### Analysis of Preparations

Limits with adjacent areas and borders between layers for GAD67-, CR- and PV-immunostained sections were established from the corresponding NeuN-immunostained section of each series. The entire coronal areas of layers I–III of the PC and of the endopiriform nucleus were measured. The border between layers I and II was usually irregular and was established according to the best fitting line. This caused an abnormal NeuN-ir neuron count in layer I (mostly excitatory) which should be included in layer II counts. The medial border between the endopiriform nucleus and the claustrum was established by prolonging the dorsal and ventral limits of the PC as is shown in the plots of Figures 1–4. In visual areas 17, 18 and 19, were measured the areas of each layer comprised in 750-μm-wide probes covering the entire thickness of these visual areas (see Figures 6B,E,H,K cited out of order here).

NeuN-ir, GAD67-ir, CR-ir and PV-ir neurons were plotted from anterior and posterior coronal sections of the PC and endopiriform nucleus, and from lateral to medial parasagittal sections of visual areas 17, 18 and 19. The relative frequencies (percentage of ir-neurons per layer with respect to total ir-neurons) was calculated in the piriform and visual cortices, and in the endopiriform nucleus to normalize the density of stained neurons (these percentages added up to 100). In addition, we also calculated the proportion of GABAergic with respect to NeuN-ir neurons. To reduce errors in the calculation of percentages and proportions, cells out of focus were not included in our counts. In the adult cerebral cortex of mammals, the proportions of GABAergic neurons(GAD67-ir, CR-ir and PV-ir) with respect to NeuN-ir neurons give a good estimation of the excitatory/inhibitory neuron ratio in a layer given that the NeuN antibody stains almost all cortical neurons (both excitatory and inhibitory), with the exception of the scarcer excitatory Cajal-Retzius neurons of layer I, which do not express NeuN (Mullen et al., 1992). The sampled area measurements and number of cells were obtained from plots using the Cellgraph system (Microptic, Barcelona, Spain).

### Deconvolution and Quantitative Measurements of Confocal Images

Two sets of sections (anterior and posterior) from four ferrets, taken at similar antero-posterior levels, were immunostained for confocal analysis. Confocal images (120 μm × 120 μm × 2 μm; thickness was obtained from one stack containing two 1-μm-thick frames). Orthogonal maximal projections were obtained, deconvoluted and analyzed with ImageJ software (the mask was set from three to eight pixels) as described elsewhere (Navarro et al., 2015). Deconvoluted confocal images showed clear VGluT1-ir and VGAT-ir boutons (see Figures 5, 6, 10, 11, 12 cited out of order here). Boutons counts, and their areas and circularity ratios [4π (bouton area)/(bouton perimeter)^2^; with a value of 1.0 indicating a perfect circle and as the value approaches 0.0, it indicates an increasingly elongated shape] were calculated by measuring the pixel area on the 2D deconvoluted image. In the piriform network, 12 confocal images randomly placed over each PC layer (six dorsal and six ventral) and endopiriform nucleus were scanned. In total, 48 anterior and 48 posterior samples per ferret were examined (this yielded a total of 384 images that were deconvoluted and analyzed). In the visual areas, six confocal images randomly placed over each visual layer were scanned, resulting in 36 analyzed samples per visual area and ferret (in total, 432 images per ferret were deconvoluted and analyzed), using the ImageJ software.

**Figure 9:**
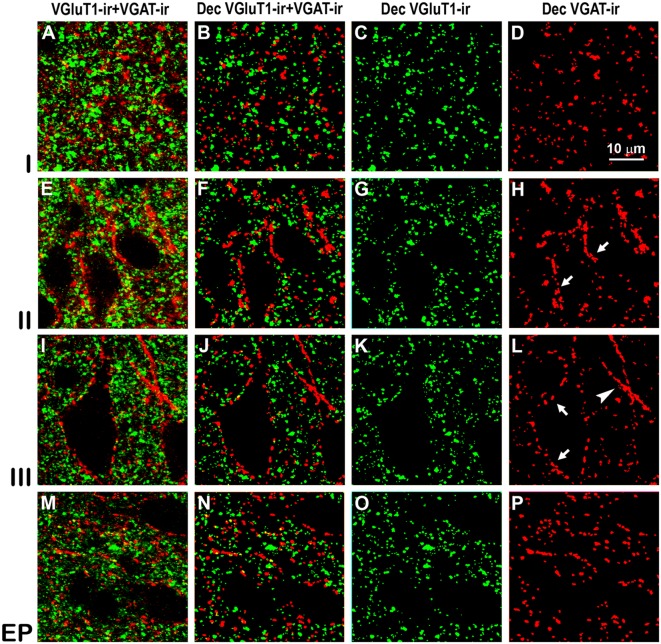
Confocal immunolabeling of VGluT1-ir and VGAT-ir boutons in the posterior piriform network. Same legend as in Figure 8.

**Figure 10:**
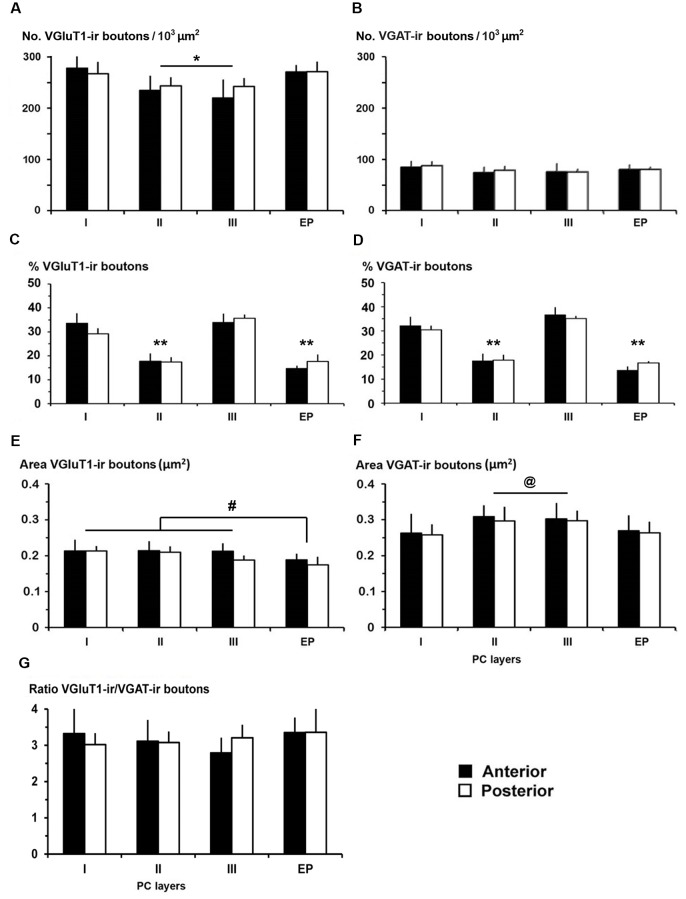
VGluT1-ir and VGAT-ir bouton distribution in the piriform network. **(A,B)** Bar charts show a higher VGluT1-ir bouton density in PC layer I and in endopiriform nucleus (EP) than in PC layers II and III. No differences were found in VGAT-ir bouton density between PC and EP. **(C,D)** The percentages of both VGluT1-ir and VGAT-ir boutons were significantly higher in PC layers I and III. **(E,F)** VGluT1-ir bouton area was lower than VGAT-ir bouton area in both PC and EP. **(E)** VGluT1-ir bouton area in EP was lower than in PC layers I–III, while **(F)** VGAT-ir bouton area decreased in PC layer I and EP. **(G)** VGluT1-ir/VGAT-ir bouton ratio shows 2.8–3.4 times more VGluT1-ir (excitatory) than VGAT-ir (inhibitory) boutons. No differences were found between anterior and posterior regions. ^#^*P* < 0.07, ^@^*P* < 0.06, *P < 0.05, **P < 0.001.

**Figure 11:**
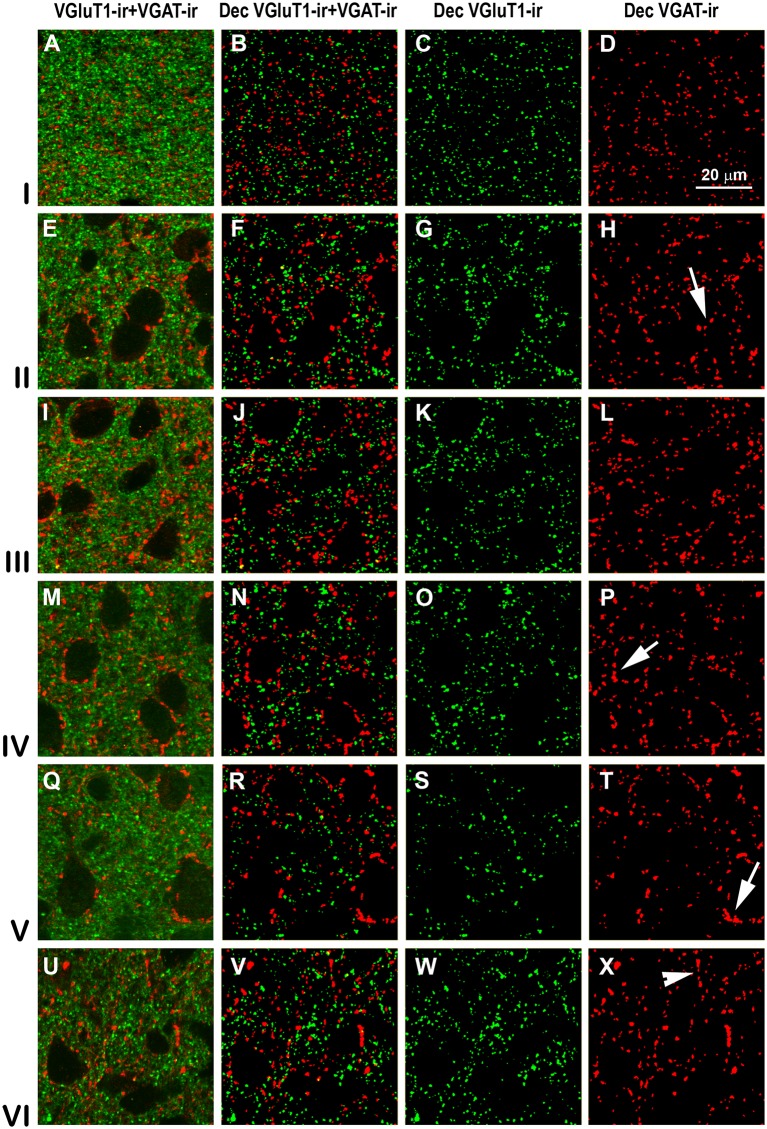
VGluT1-ir and VGAT-ir boutons in primary visual area 17. **(A,E,I,M,Q,U)** Confocal high-power photomicrographs, taken perpendicular to the pial surface, show VGluT1-ir (green) and VGAT-ir (red) boutons for each cortical layer (left). **(B,F,J,N,R,V)** Deconvoluted merged images (Dec VGluT1-ir/VGAT-ir). **(C,G,K,O,S,W)** Deconvoluted images show VGluT1-ir bouton profiles (Dec VGluT1-ir). **(D,H,L,P,T,X)** Deconvoluted images show VGAT-ir bouton profiles (Dec VGAT-ir). Perisomatic (arrows) and chandelier-like (arrowhead) VGAT-ir boutons are indicated. Same scale for all images.

**Figure 12:**
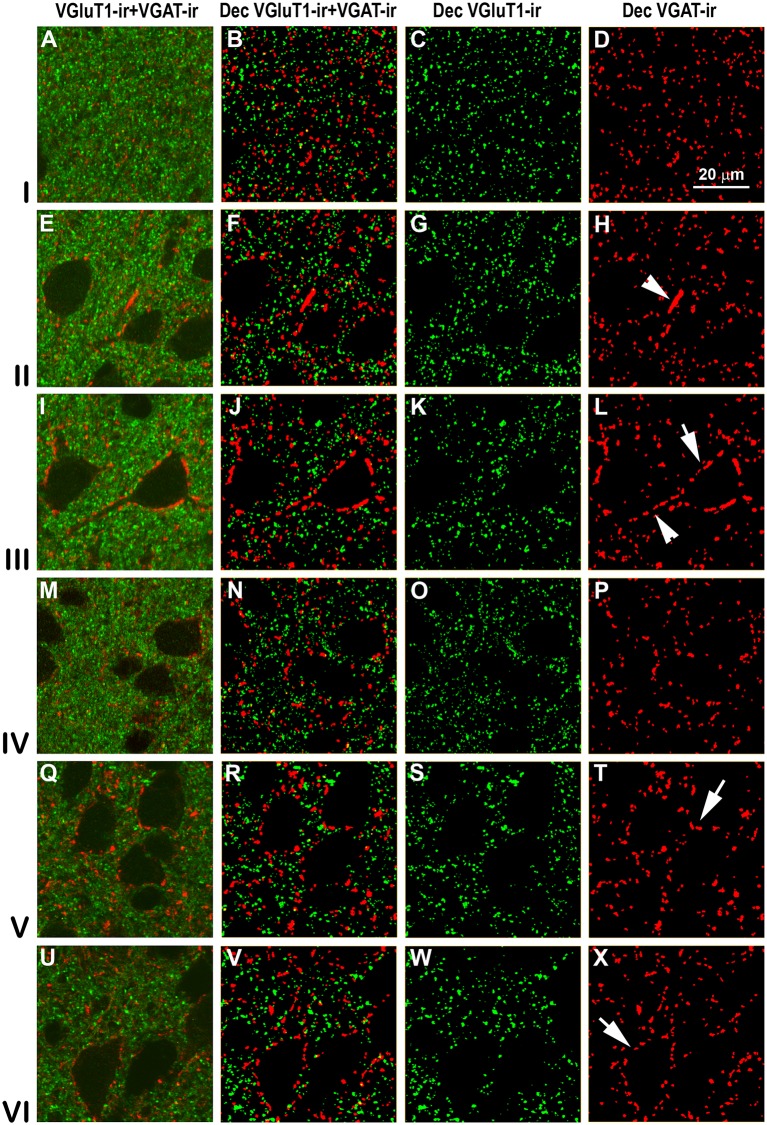
VGluT1-ir and VGAT-ir boutons in secondary visual area 18. Same legend as in Figure 11.

### Statistical Analysis

For statistical analysis, we used the SYSTAT software (Systat Software, Inc., Chicago, IL, USA). Frequency distributions of immunostained cells and of VGluT1-ir and VGAT-ir boutons were analyzed using one and two-way ANOVAs followed by either Tukey’s (equal variances) or Games-Howell’s (unequal variances) tests to identify significant differences (*P* < 0.05) in means between piriform and visual layers, and between piriform antero-posterior regions and visual areas. In order to compare means, the densities of immunostained cells were adjusted to equivalent areas of 10^5^ μm^2^ and those of immunostained boutons to equivalent areas of 10^3^ μm^2^. All bar histograms show mean ± SD.

## Results

### Cellular Organization of the Piriform Network

The dorsal and ventral limits of the PC with adjacent cortical areas were established according to previous reports (Ramón y Cajal, 1911; O’Leary, 1937; Valverde, 1965; Stevens, 1969; Haberly, 1983). In the ferret, as in other vertebrates, the thickness of layers II–III was the major cytoarchitectonic difference between the PC and adjacent cortical areas. These limits were well defined in horizontal and coronal NeuN-ir sections (Figures 1A,C–E). In cytochrome oxidase-stained sections, layer II and the superficial tier of layer III in the PC were also more densely stained than the adjacent areas (Figures 1I,J). In contrast, the borders of the endopiriform nucleus were more difficult to establish. Dorsally, laterally and ventrally, they were clearly distinguishable since the cell density was higher in the endopiriform nucleus than in the adjacent PC layer III, while the medial border with the claustrum was blurred (Figures 1A,C,D).

In both horizontal (Figure 1A) and coronal (Figures 1C,D,E) NeuN-ir sections, layers were well delineated. The lowest density of neurons was observed in PC layer I. Layer II showed pyramidal-like and round cell bodies (mostly located in layer IIb) more densely packed and smaller in size than in the adjacent layer III (Figure 1E). In the superficial part of layer III, many neurons showed pyramidal-like and multipolar somata and were more densely packed than in the deep part where, in addition to pyramidal-like and multipolar neurons, many fusiform neurons could be seen. In the endopiriform nucleus, round and fusiform neurons were seen more densely packed than in the adjacent PC layer III (Figure 1E).

On average, the percentage of NeuN-ir neurons in the piriform network (anterior and posterior regions pooled together) was 42.9 ± 4.3% in layer II, and significantly less in layer III (31.5 ± 4.0%), endopiriform nucleus (22.5 ± 3.6%) and layer I (3.1 ± 0.8%; *P* < 0.001; Figures 1F,G). The percentage of NeuN-ir neurons in the posterior PC sections was significantly lower in layer II and higher in layer III than in the anterior counterparts (*P* < 0.05; Figure 1G; Supplementary Table S1).

### GABAergic (GAD67-ir, CR-ir and PV-ir) Neurons in the Piriform Network

Subsets of GABAergic neurons were investigated by GAD67, CR and PV immunostaining. In the PC and endopiriform nucleus, most GAD67-ir neurons resembled bipolar- or multipolar-like cells (small and large sized). In PC layers II–III, there were numerous processes and boutons (Figures 2A–D), and in the piriform network, numerous perisomatic boutons could be seen (Figures 2D,E). On average, the percentage of GAD67-ir neurons in the piriform network was 55.4 ± 3.8% in layer III, and significantly less in the endopiriform nucleus (18.3 ± 2.4%), layer II (16.6 ± 1.8%) and layer I (9.6 ± 1.7%; *P* < 0.001; Figures 2F–H). No significant differences were found between the anterior and posterior levels.

Dendrites and axons of CR-ir and PV-ir neurons were partially stained. However, many CR-ir neurons resembled bipolar-like cells with processes perpendicular to the pial surface in PC layer II; they were small and large multipolar-like in layer III, and multipolar-like or fusiform bipolar-like in the endopiriform nucleus (Figures 3A–D). In the endopiriform nucleus, there were numerous boutons in the neuropil and more scarcely around unstained cell bodies (Figure 3E). On average, the percentage of CR-ir neurons was 34.4 ± 3.9% and 38.1 ± 4.2% in PC layers II–III, respectively, and significantly less in the endopiriform nucleus (21.9 ± 4.1%) and PC layer I (5.5 ± 1.6%; *P* < 0.001; Figures 3F–H). No significant differences were found between the anterior and posterior levels.

PV-ir neurons showed variable sizes and resembled mostly multipolar-like cells (Figures 4A–D). Numerous boutons around unstained neuronal somata were observed especially in PC layers II–III (Figures 4D,E). PV-ir chandelier-like boutons (DeFelipe et al., 1989) were scarce in the piriform network. On average, the percentage of PV-ir neurons was 47.2 ± 6.7% in layer III, and significantly less in PC layers I (15.7 ± 1.9%) and II (22.8 ± 7.0%) and in the endopiriform nucleus (14.3 ± 4.0%; *P* < 0.001; Figures 4F–H). The percentage of PV-ir neurons was higher in posterior (17.4 ± 4.7%) than in anterior (10.9 ± 2.3%) regions of the endopiriform nucleus (*P* < 0.05; Figure 4H).

### Architectonic Organization of Visual Areas 17, 18 and 19

Published descriptions of cortical layer thickness and cell morphology coincided with our observations for areas 17 (Rockland, 1985; Law et al., 1988; Innocenti et al., 2002), and 18 and 19 (Innocenti et al., 2002). The limits between visual areas 17, 18 and 19 were established in Nissl- and cytochrome oxidase-stained sections, and in NeuN-ir sections (Figures 5B, 6A), following already published criteria (Innocenti et al., 2002). The limits between layers in GAD67-, CR- and PV-immunostained sections were established from adjacent NeuN-ir sections. In all visual areas, the border between layer VI and the adjacent subcortical white matter was usually irregular and was established according to the best fitting line. Sulci and gyri regions were excluded from the plots.

NeuN-ir neuron density was greater in area 17 (141 ± 32 neurons/10^5^ μm^2^; *P* < 0.05) than in areas 18 and 19 (119 ± 20 and 104 ± 18 neurons/10^5^ μm^2^, respectively). NeuN-ir neuron percentage in layer IV relative to the other layers was higher in area 17 (27.7 ± 2.8%) than in areas 18 and 19 (on average, 18.8 ± 1.9%, *P* < 0.001; Figures 5C, 6B,C; Supplementary Table S2).

### GABAergic (GAD67-ir, CR-ir and PV-ir) Neurons in Visual Areas 17, 18 and 19

The distribution and morphology of GAD67-ir, CR-ir and PV-ir neurons were similar to those described in other vertebrates (see review by DeFelipe, 1997). In summary, in all areas, they were very scarce in layer I, both GAD67-ir and PV-ir neurons were mostly distributed in layers II–VI, while CR-ir neurons were densest in layers II–III and progressively less dense in layers IV–VI (Figures 5D–G, 6D,E,G,I). Many CR-ir neurons resembled bipolar-like cells with processes perpendicular to the pial surface. Numerous CR-ir axon-like processes and boutons were observed in layers I–II (Figures 5F,G). PV-ir neurons showed large- and medium-sized soma localized in layers II–VI, and resembled multipolar-like cells. Perisomatic and chandelier-like PV-ir boutons were seen in layers II–VI (Figure 5I; Supplementary Figure S1).

The GAD67-ir neuron percentage with respect to the other layers was highest in layers III–IV of area 17 (on average, 28.6 ± 3.2%) and lower in layers II and V–VI (on average, 14.1 ± 1.4%; *P* < 0.001; Figure 6F; Supplementary Table S1). In areas 18 and 19, it was highest in layer III (on average, 28.9 ± 3.0%) and lower in layers II and IV–VI (on average, 17.6 ± 1.9%; *P* < 0.001). Compared to areas 18 and 19, GAD67-ir neuron percentage in area 17 was significantly higher in layer IV (26.8 ± 3.2% in area 17 vs. 20.4 ± 2.9% on average in areas 18 and 19; *P* < 0.01) and lower in layer VI (14.3 ± 0.6% in area 17 vs. 19.7 ± 2.1% on average in areas 18 and 19; *P* < 0.05; Figure 6F; Supplementary Figure S2, Table S2).

In all areas, CR-ir neuron percentage (layer I excluded) was highest in layers II–III (on average, 35.8 ± 3.2%) and progressively decreased (*P* < 0.001) in layers IV (15.5 ± 1.7%) and V–VI (on average, 5.9 ± 1.0%). In layer IV, CR-ir neuron percentage was higher in area 17 (17.9 ± 1.9%) than in areas 18 and 19 (on average, 7.5 ± 1.5%; *P* < 0.05; Figure 6I; Supplementary Figure S2, Table S2).

In area 17, PV-ir neuron percentage (layer I excluded) was highest in layer IV (37.4 ± 3.1%), lower in layer III (24.4 ± 2.3%) and even lower in layers II, V–VI (on average, 13.4 ± 1.6%; *P* < 0.001; Figure 6L). In contrast, in areas 18 and 19, PV-ir neuron percentage was highest in layers III and VI (on average, 26.4 ± 2.1%) and lower in layers II, IV–V (on average, 15.3 ± 1.4%; *P* < 0.001; Figure 6L, Supplementary Figure S2, Table S2). PV-ir neuron percentage in layer IV was higher in area 17 (37.4 ± 3.1%) than in areas 18 and 19 (on average, 14.7 ± 1.2%; *P* < 0.001), and in layer VI was lower in area 17 (12.7 ± 1.2%) than in areas 18 and 19 (on average, 25.0 ± 1.7%; *P* < 0.001; Figure 6L; Supplementary Figure S2, Table S2).

As an approximation of the excitatory and inhibitory balance in visual areas, we first computed the proportion of GABAergic neurons with respect to the total number of NeuN-ir neurons (Figures 6M–O, Supplementary Table S2). On average, 33.6 ± 2.6% of all neurons per layer were GAD67-ir in layers II-V of area 17. The proportion of GAD67-ir neurons decreased in layer VI (25.7 ± 2.5%) and was very low in layer I (5.8 ± 2.1%; Figure 6M). In area 18, layer V had the highest GAD67-ir neuron proportion (36.7 ± 3.3%), followed by layers II and IV (on average, 32.1 ± 1.5%) and was lowest in layers I and VI (on average, 22.7 ± 1.0%). Area 19 showed a somewhat similar pattern to area 17 where 27.3 ± 2.3% of GAD67-ir neurons were in layers II–V, and 17.3 ± 2.3% in layers I and VI (Figure 6M; Supplementary Table S2).

CR-ir neuron proportion was highest in layer II of all areas (on average, 34.4 ± 1.2%), followed by layer III (on average, 20.3 ± 1.0%), layers IV–V (on average, 9.3 ± 0.9%) and layer VI (3.2 ± 0.4%). In layer I, CR-ir neuron proportion increased from on average 6.8 ± 1.4% in areas 17 and 18–18.7 ± 1.4% in area 19 (Figure 6N; Supplementary Table S2).

In area 17, PV-ir neuron proportion was highest in layers IV–V (on average, 22.0 ± 2.9%), followed by layers II–III and VI (on average, 15.6 ± 2.3) and finally by layer I (4.6 ± 1.6%). In areas 18 and 19, PV-ir neuron proportion was highest in layers I and V of areas 18 and 19 (on average, 20.1 ± 1.5%), followed by all other layers (on average, 13.6 ± 1.5; Figure 6M; Supplementary Table S2).

### Comparative Cellular Organization Between Piriform Network and Visual Areas 17, 18 and 19

The percentage of GAD67-ir neurons in layer III was similar between the PC (67.9 ± 4.7%) and visual areas (on average, 66.0 ± 6.1%). However, it was lower (*P* < 0.001) in PC layer II (20.4 ± 2.5%) than in visual areas (on average, 32.7 ± 3.1%), and higher (*P* < 0.001) in PC layer I (11.8 ± 1.6%) than in visual areas (on average, 1.3 ± 0.6%; Figure 7A).

Likewise, the distribution of CR-ir and PV-ir neurons across layers I–III was similar between the PC and visual cortices, even though the percentage of CR-ir neurons in layer I was higher in the PC (7.1 ± 2.0%) than in visual areas (1.3 ± 1.2%; *P* < 0.001). There were no differences in layer II CR-ir neuron percentage between PC (44.1 ± 5.5%) and visual areas (on average, 43.8 ± 4.4%), and in layer III CR-ir neuron percentage between PC (48.8 ± 5.6%) and visual areas (on average, 55.0 ± 5.4%; Figure 7B).

Similarly, the percentage of PV-ir neurons in layer I was higher in the PC (18.3 ± 3.4%) than in visual areas (2.6 ± 1.1%; *P* < 0.001) but was lower in PC layers II (26.6 ± 6.6%) and III (55.1 ± 8.1%) than in the visual counterparts (33.1 ± 4.0% and 64.3 ± 6.2%, respectively; *P* < 0.05; Figure 7C). There were no differences in GAD67-ir, CR-ir and PV-ir neuron percentage in layers I–III between anterior and posterior PC regions (Figures 2H, 3H, 4H, 7A–D).

The proportions of GAD67-ir, CR-ir and PV-ir neurons with respect to the total number of NeuN-ir neurons were calculated and contrasted first for layers I–III in piriform and visual cortices, and later for the piriform network (piriform layers I–III and endopiriform nucleus) and layers I–VI of visual areas (Figures 7D–G).

The GABAergic population was significantly lower in layers I–III of the PC than in layers I–III of the visual areas. The averaged proportion of GAD67-ir with respect to NeuN-ir neurons was 15.3% in the PC vs. 29.3% in visual areas (*P* < 0.001; Figure 7D). There were 10.9% CR-ir neurons in the PC and 24.3% in visual areas (*P* < 0.001; Figure 7D). More importantly, there were 51.6% fewer PV-ir neurons in the PC than in visual areas (7.4% in PC vs. 14.8% in visual areas; *P* < 0.001; Figure 7D).

Considering the piriform network and layers I–VI of the visual areas, the proportion of GABAergic neurons with respect to the total number of neurons (NeuN-ir) was lower in the piriform network, particularly GAD67-ir and PV-ir neurons. In total, there were 14.5% GAD67-ir neurons in the piriform network vs. 28.9% in visual areas (*P* < 0.001; Figure 7E), 10.8% CR-ir neurons in the piriform network vs. 14.5% in visual areas (*P* < 0.05; Figure 7F) and 6.9% PV-ir neurons in the piriform network vs. 15.6% in visual areas (*P* < 0.001; Figure 7G).

### Distribution and Size of VGluT1-ir and VGAT-ir Boutons in the Piriform Network

High power confocal micrographs show details of raw (Figures 8A,E,I,M, 9A,E,I,M) and deconvoluted (Figures 8B–D,F–H,J–L,N–P, 9B–D,F–H,J–L,N–P) images of VGluT1-ir and VGAT-ir boutons in anterior (Figure 8) and posterior (Figure 9) regions of the PC, and in the endopiriform nucleus. In layers II–III, numerous perisomatic (Figures 8, 9, arrows) and chandelier-like boutons (Figures 8, 9, arrowheads) can be seen. These boutons were scarcer in the endopiriform nucleus.

The distribution of VGluT1-ir and VGAT-ir boutons was similar between anterior and posterior piriform network regions but did differ across layers. On average, VGluT1-ir bouton density was lower (*P* < 0.05) in layers II–III (on average, 234 ± 34 boutons/10^3^ μm^2^) than in layer I and endopiriform nucleus (on average, 271 ± 47 boutons/10^3^ μm^2^; Figure 10A; Supplementary Table S3), whilst VGAT-ir bouton density did not differ between PC layers and endopiriform nucleus (on average, 80 ± 9 boutons/10^3^ μm^2^; Figure 10B). Despite these small differences in bouton density across layers, the percentage of both VGluT1-ir and VGAT-ir boutons was lower in layers II–III than in layer I and endopiriform nucleus (*P* < 0.001; Figures 10C,D). In the piriform network, the average size of VGluT1-ir boutons (0.19 ± 0.03 μm^2^) was smaller than that of VGAT-ir boutons (0.28 ± 0.03 μm^2^; *P* < 0.001; Figures 10E,F). Additionally, the average VGluT1-ir bouton size was smaller in the endopiriform nucleus (0.18 ± 0.03 μm^2^) than in the PC (0.21 ± 0.03 μm^2^; *P* < 0.07; Figure 10E) and the VGAT-ir bouton size was smaller in PC layer I and endopiriform nucleus (on average, 0.26 ± 0.03 μm^2^) than in layers II–III (on average, 0.30 ± 0.03 μm^2^; *P* < 0.06; Figure 10F; Supplementary Table S3). In the piriform network, the average VGluT1-ir bouton circularity (0.89 ± 0.02) was significantly higher (*P* < 0.001) than that of VGAT-ir boutons (0.64 ± 0.04), showing that VGAT-ir boutons show a more elongate projected shape. VGluT1-ir and VGAT-ir bouton circularity was similar among layers and regions (Supplementary Table S3).

Whereas, the distribution of VGluT1-ir (excitatory) and VGAT-ir (inhibitory) boutons was similar across the piriform network (compare Figures 10C,D), the density of VGluT1-ir boutons was much higher than that of VGAT-ir boutons (compare Figures 10A,B). There were 2.8–3.4 times more VGluT1-ir boutons than VGAT-ir ones (Figure 10G; Supplementary Table S3). Note, however, that VGluT1-ir bouton size was smaller than VGAT-ir bouton size (compare Figures 10E,F).

### Distribution and Size of VGluT1-ir and VGAT-ir Boutons in Visual Areas 17, 18 and 19

**Figure 13:**
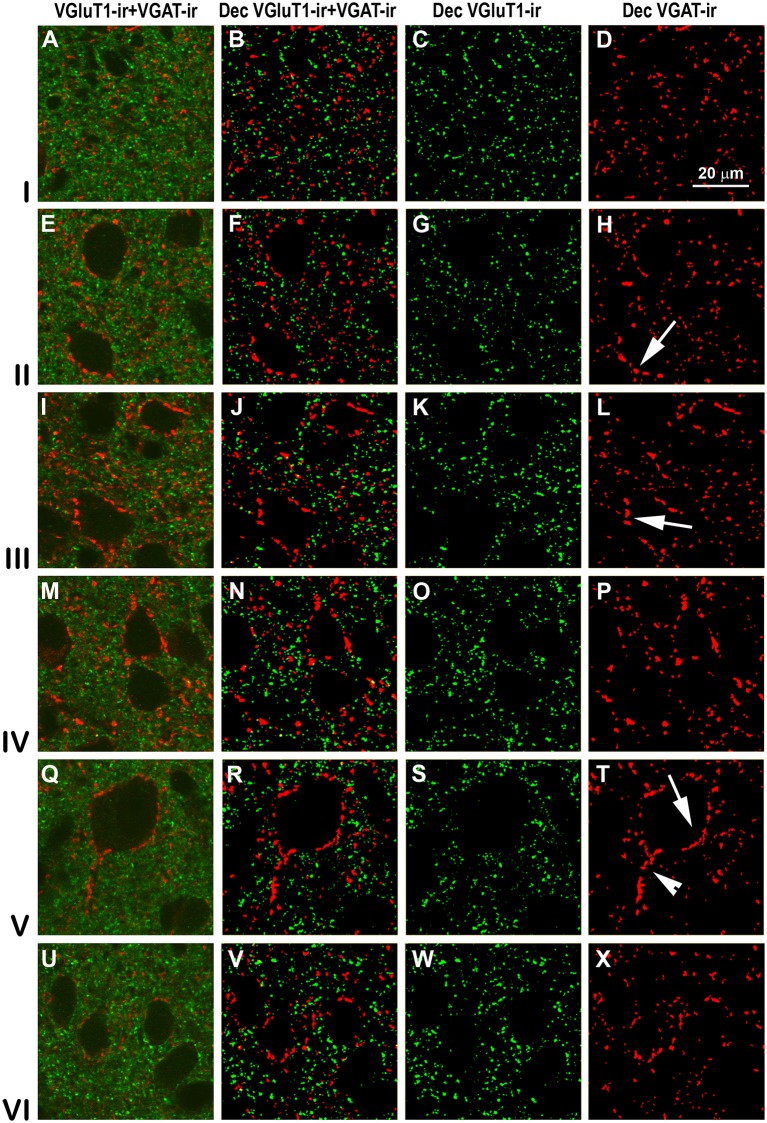
VGluT1-ir and VGAT-ir boutons in secondary visual area 19. Same legend as in Figure 11.

High power confocal micrographs of areas 17, 18 and 19 (Figures 11–13, respectively) show details of raw (VGluT1-ir+VGAT-ir column) and deconvoluted (Dec VGluT1-ir+VGAT-ir, Dec VGluT1-ir and Dec VGAT-ir columns) ir boutons. In visual layers II–VI, numerous perisomatic (Figures 11–13, arrows) and chandelier-like boutons can be seen (Figures 11–13, arrowheads). These formations were not seen in layer I.

**Figure 14:**
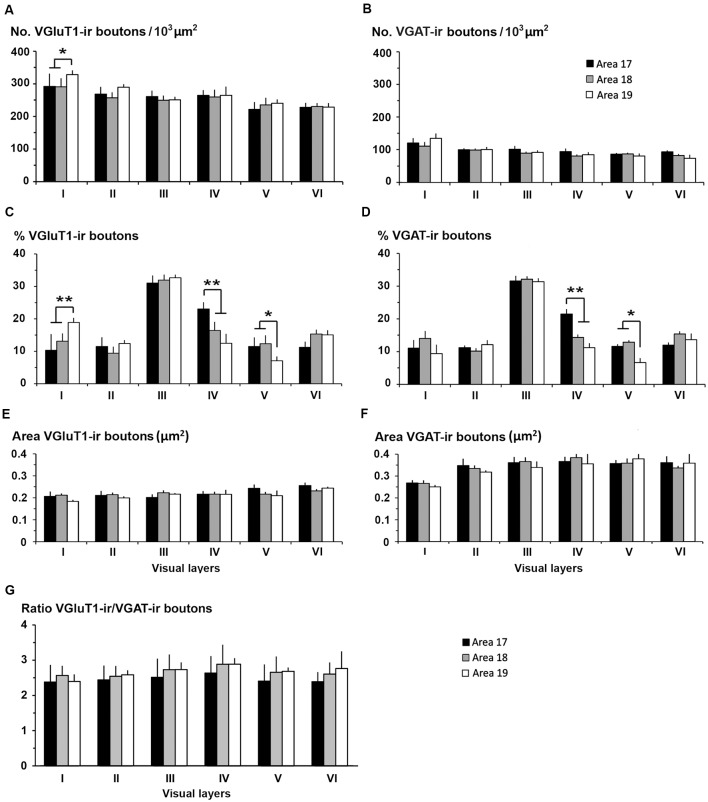
VGluT1-ir and VGAT-ir bouton distribution in visual areas 17, 18 and 19. **(A)** Bar histograms show higher VGluT1-ir bouton density in layer I of area 19. **(B)** No differences were found in VGAT-ir bouton density between or within visual areas. **(C)** VGluT1-ir bouton percentage per layer was higher in layer I of area 19, in layer IV of area 17, and in layer V of areas 17 and 18. **(D)** VGAT-ir bouton percentage was higher in layer IV of area 17 and lower in layer V of area 19. **(E,F)** VGluT1-ir bouton area was smaller than the VGAT-ir bouton area. **(G)** VGluT1-ir/VGAT-ir bouton ratio in the visual areas showed on average 2.6 ± 0.4 times more excitatory than inhibitory boutons. No differences were found between layers. Vertical lines in bar histograms show SD. *P < 0.05, **P < 0.001.

In all visual areas, VGluT1-ir and VGAT-ir bouton density progressively decreased from layer I (303 ± 29 VGluT1-ir boutons/10^3^ μm^2^ and 123 ± 78 VGAT-ir boutons/10^3^ μm^2^) to layer VI (219 ± 22 VGluT1-ir boutons/10^3^ μm^2^ and 84.7 ± 6.9 VGAT-ir boutons/10^3^ μm^2^; Figures 14A,B, Supplementary Table S4). VGluT1-ir bouton density was highest in layer I of area 19 (328 ± 32 boutons/10^3^ μm^2^; *P* < 0.05). Despite the relatively small difference in density across layers, in all visual areas, VGluT1-ir and VGAT-ir bouton percentage was highest in layer III (33.0 ± 3.0 and 30.0 ± 2.4%, respectively) due to its larger thickness (Figures 14C,D, Supplementary Table S4). VGluT1-ir and VGAT-ir bouton percentage in layer IV was higher in area 17 (22.7 ± 3.3 and 21.4 ± 2.5%, respectively) than in areas 18 and 19 (on average, 13.8 ± 1.2 and 12.8 ± 1.1%, respectively; *P* < 0.001; Figures 14C,D; Supplementary Table S4).

VGluT1-ir bouton area was slightly larger, although not significantly, in layers V–VI of area 17 (on average, 0.25 ± 0.03 μm^2^) than in the other layers and areas (on average, 0.22 ± 0.03 μm^2^). In all areas, VGAT-ir bouton area was lower in layer I (0.26 ± 0.01 vs. 0.36 ± 0.03 μm^2^ on average in layers II–VI; *P* < 0.05; Figures 14E,F, Supplementary Table S4). The average VGluT1-ir bouton circularity (0.89 ± 0.01) was significantly higher (*P* < 0.001) than that of VGAT-ir boutons (0.63 ± 0.05), showing that VGAT-ir boutons show a more elongate projected shape. VGluT1-ir and VGAT-ir bouton circularity was similar among layers and visual areas (Supplementary Table S4). It is worth noting that, even though the density of VGluT1-ir boutons was much higher than that of VGAT-ir boutons, VGAT-ir bouton area was on average 1.54 times larger than that of VGluT1-ir boutons (Figures 14E,F, Supplementary Table S4).

An approximation of the balance between excitatory and inhibitory inputs was better estimated from the ratio of VGluT1-ir to VGAT-ir boutons. On average, the VGluT1-ir/VGAT-ir bouton ratio was lower in area 17 (2.5 ± 0.4) than in areas 18 and 19 (2.7 ± 0.3; *P* < 0.05). In all areas, VGluT1-ir boutons surpassed VGAT-ir ones by 2.6 ± 0.4 times (Figure 14G, Supplementary Table S4).

### Comparative Distribution of Excitatory and Inhibitory Boutons Between the Piriform Network and Visual Areas 17, 18 and 19

**Figure 15:**
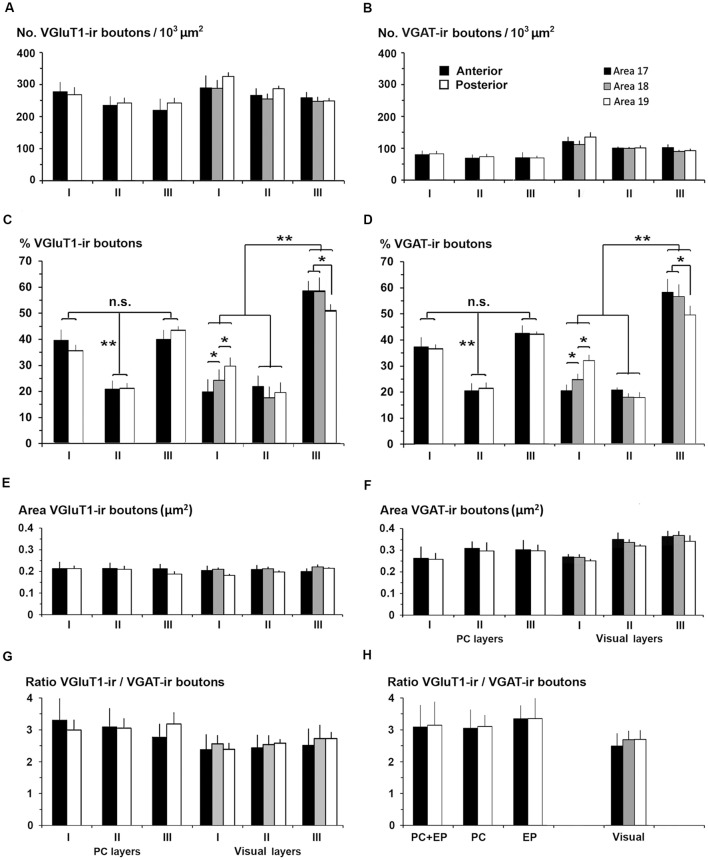
VGluT1-ir and VGAT-ir bouton distribution between the piriform network and visual areas 17, 18 and 19. **(A,B)** Bar charts show that both VGluT1-ir and VGAT-ir bouton densities were slightly lower in layers I–III of the piriform cortex (PC) than of visual areas, and that both VGluT1-ir and VGAT-ir bouton percentage was lowest (*P* < 0.001) in PC layer II **(C,D)** while in visual areas, both were lower (*P* < 0.001) in layers I and II (**C,D**). No differences were found in VGluT1-ir bouton areas **(E)** although it was lower than the corresponding VGAT-ir bouton area (compare **E** with **F**). VGAT-ir bouton area in PC layers II–III was lower than in visual areas (**F**; *P* < 0.05). No further significant differences were found. **(G)** VGluT1-ir/VGAT-ir bouton ratio was higher in PC than in visual layers I–III. **(H)** VGluT1-ir/VGAT-ir bouton ratio in PC, endopiriform nucleus (EP) and piriform network (PC+EP) was also higher than in layers I–VI of visual areas. n.s., not significant differences, *P < 0.05, **P < 0.001.

We compared the excitatory/inhibitory profile of the piriform network with that of the visual areas to identify some potential explanation for the differences in excitability and propagation speed between the two. VGluT1-ir and VGAT-ir bouton density was on average lower in PC layers I–III than in visual layers I–III (247 ± 34 VGluT1-ir boutons/10^3^ μm^2^ in PC vs. 275 ± 28 boutons/10^3^ μm^2^ in visual areas; *P* < 0.05; Figure 15A; and 80 ± 10 VGAT-ir boutons/10^3^ μm^2^ in PC vs. 108 ± 6 boutons/10^3^ μm^2^ in visual areas; *P* < 0.05; Figure 15B). VGluT1-ir and VGAT-ir bouton percentage across layers I–III was similar in layers II of PC and visual areas, while it was higher in PC layer I, and lower in PC layer III. The size of VGluT1-ir boutons was similar between layers I–III of the PC and visual areas (0.209 ± 0.02 vs. 0.212 ± 0.02 μm^2^, respectively; Figure 15E), while that of VGAT-ir was smaller in PC layers II–III (on average, 0.29 ± 0.03 μm^2^) than in their visual counterparts (on average, 0.32 ± 0.02 μm^2^; *P* < 0.05; Figure 15F). Except for bouton percentage (Figures 15C,D), no differences were found across layers I–III neither between anterior and posterior PC regions nor across visual areas 17, 18 and 19 (Figures 15A,B,E–H).

The VGluT1-ir/VGAT-ir bouton ratio in the piriform network, regardless of whether only layers I–III or the whole network was taken into account, was higher than the visual areas. VGluT1-ir/VGAT-ir bouton ratio was 3.1 in PC layers I–III vs. 2.6 in their visual counterparts (Figure 15G), and 3.2 in the piriform network vs. 2.6 in visual layers I–VI (Figure 15H).

## Discussion

As mentioned previously, our aim was to provide a better understanding of the excitatory/inhibitory system in the piriform network. Our findings show that GAD67-ir neuron proportion was 14.6% in the piriform network; it was 31.1% in primary visual area 17, and 29.4 and 24.6% in secondary areas 18 and 19, respectively. CR-ir neuron proportion was 10.8% in the piriform network vs. 14.5% in visual areas 17, 18 and 19 (no significant differences between areas were found). PV-ir neuron proportion was 6.7% in the piriform network; it was 17.9% in area 17 and decreased in areas 18 and 19 (on average, 14.4%; Figures 7E–G). In all visual areas, there were 2.6 ± 0.4 times more VGluT1-ir than VGAT-ir boutons, this ratio increased to 3.2 ± 0.4 in the piriform network, which represents a 18.8% increase (Figure 15H).

These data reveal that the distribution of relevant GABAergic neurons, involved in cortical excitability and the density and size of VGluT1-ir and VGAT-ir boutons in layers I–III are similar between the piriform and visual cortices. However, the percentage of GABAergic neurons with respect to the total number of neurons was lower, and the VGluT1-ir/VGAT-ir bouton ratio higher, in the PC with respect to visual layers I–III. These findings reflect decreased inhibition in the piriform network compared with the visual cortex and are relevant to better understand the high excitability of the piriform network.

### Technical Considerations

The accurate distribution of GABAergic neurons in the ferret cortex was accomplished by a correct discrimination of cortical layers. Cytoarchitectonic criteria provided by Nissl and cytochrome oxidase staining helped to identify cortical areas and layers, but area and layer limits were clearer in NeuN-ir sections. The percentage of NeuN-ir neurons and the proportion of GABAergic neurons with respect to NeuN-ir neurons can be affected by the delineation of the limits. This was particularly relevant in the PC (Figures 1C–E) due to the often irregular border between layers I and II. Thus NeuN-ir neuron counts in layer I (mostly excitatory) very likely include neurons of layer II. However, the percentage of NeuN-ir neurons in layer I was very low compared to the percentages in the other layers (on average, 3.25% in the piriform network and 1.47% in visual areas; Supplementary Tables S1, S2, respectively).

We computed the distribution of NeuN-ir neurons and three types of GABAergic neurons (GAD67-ir, CR-ir and PV-ir) across layers in each visual area. As discussed below the classification of inhibitory neurons is a very complex issue (Ekstrand et al., 2001a; DeFelipe et al., 2013; Taniguchi, 2014). We immunostained the three major subtypes of GABAergic neurons. Among these, PV-ir neurons are considered the most important cell type controlling the excitatory output of pyramidal neurons (Somogyi et al., 1982) and are involved in epilepsy in humans (DeFelipe, 1999). Owing to the limitations of immunostaining which result in incomplete labeling of processes, our morphological descriptions are based on the size and shape of the soma, and on the morphology of the proximal dendritic segments and axons (including boutons). In addition, in the parasagittal sections of the ferret visual cortex, the curvature of the cortex increases in the lateral sections, thus some axon initial segments might appear at a different plane of their parent soma, resulting in isolated PV-ir chandelier-like boutons (Supplementary Figure S1). In this study, we have not performed double GAD67 and CR or PV immunostaining. However, each immunostain was carried out in consecutive sections and the proportion of GABAergic-ir neurons to NeuN-ir neurons was calculated with respect to the same NeuN immunostained section. As such, the respective layer densities of CR-ir and PV-ir neurons cannot be added to obtain a GAD67-ir neuron density. In some layers, GAD-ir neuron percentage was similar to the PV-ir neuron percentage. This reflects the dependence of GAD67-ir immunostaining on the degree of GAD67 expression and neuron size (Hendrickson et al., 1994). We have seen both heavy and light GAD67 immunostained neurons. Lighter stained neurons may be masked by the background level of stain, contributing in part, to the unexpected GAD67-ir percentages in some cortical layers. However, despite the possible loss in the counting of GAD67-ir neurons, the laminar distribution of GAD67-ir neurons remains useful and gives an overall view of the cortical distribution of GABAergic neurons.

A recent study using a Gaussian filter and an automated object detection process reported a new approach to a fast and accurate 3D analysis of fluorescent puncta in the neuropil of large brain areas, avoiding the more tedious deconvolution procedure (Varando et al., 2018). In our study, we used the deconvolution procedure for the analysis of 2D confocal images, which gives also an accurate bouton quantification to define the inhibitory profile of the ferret visual cortex. Our findings show that VGluT1-ir boutons clearly outnumber the VGAT-ir ones by 2.6 ± 0.4 times in visual areas and 3.2 ± 0.4 times in the piriform network. However, independently of the procedure used in confocal microscopy, VGluT1-ir/VGAT-ir bouton ratio should be interpreted cautiously since the number of VGluT1-ir and VGAT-ir boutons that establish active synapses on target neurons in the ferret cortex remains unknown. The VGluT1-ir/VGAT-ir bouton ratio (2.6 ± 0.4) that we found in visual areas is significantly lower than that found in scanning electron microscope (SEM) studies (ca 20:1; Kasthuri et al., 2015) showing a ratio between excitatory/inhibitory synapses of 20.2 on dendrites of excitatory neurons and 9.7 on inhibitory dendrites. These differences might be due to an overestimation of the excitation/inhibition in the cerebral cortex, since: (i) the SEM study covered a very small fraction of the cortical volume (40 μm × 40 μm × 50 μm); (ii) this ratio may vary from one layer to another, and between species; and (iii) the classification of synapses was based on the presynaptic characteristics, which may make it difficult to differentiate between excitatory and inhibitory synapses. Despite the possible overestimation of the Kasthuri et al.’s (2015) study, our counts are an underestimation because, regardless of the confocal procedure used, the smallest fluorescent boutons are masked by the fluorescent background of the sections inherent to the methodology. This may especially affect the VGluT1 boutons because they are smaller than the VGAT-ir ones. Furthermore, the VGluT2 glutamatergic boutons were not labeled in our study. In the cerebral cortex, VGluT1 and VGluT2 antibodies label two separate sets of boutons. While VGluT1-ir boutons are more widely distributed and synapse on pyramidal neurons among all cortical layers, VGluT2-ir boutons are found in sensory thalamic afferents and are mostly localized in layer IV of sensory neocortical areas (Fremeau et al., 2001; Fujiyama et al., 2001). Also, since in layers I–III the density of VGluT2-ir boutons is very low both in visual and piriform cortices, and sensory thalamic afferents target only visual areas, in the present study only VGluT1-ir labeling was used. Therefore, the VGluT1 and VGAT immunolabeling should be a good approach for the comparison of the VGluT1-ir/VGAT-ir bouton ratio in layers I–III in ferret piriform and visual cortices, reflecting a good estimation of the excitation/inhibition input balance in the piriform network (Chaudhry et al., 1998; Minelli et al., 2003a,b; Alonso-Nanclares et al., 2004). However, for a more accurate approximation of the excitatory/inhibitory input in the studied areas, further studies should combine electron microscopy and electrophysiological methods.

### Cellular Organization of the Piriform Network

As already mentioned, the limits of the areas in the piriform network were localized following the nomenclature and layer division established in rats, opossums and cats (Haberly, 1983, 1990; Witter et al., 1988; Ekstrand et al., 2001b; Neville and Haberly, 2004; Larriva-Sahd, 2010). The major discrepancy with other studies (for discussion, see Haberly, 1983) was that several authors considered the most caudal posterior PC a portion either of the periamygdaloid complex (O’Leary, 1937) or of the sphenoidal cortex (Ramón y Cajal, 1911). In the PC, the cytoarchitectonic features in the anterior and posterior regions were similar, showing a well-defined three-layered structure, and the limits with adjacent cortical areas, as well as with the endopiriform nucleus, were clear. The relationship between the PC and endopiriform nucleus in mammals remains under discussion because while the dorsal, lateral and ventral limits of the endopiriform nucleus with the adjacent PC are clear cut, the medial limit with the claustrum is blurred and difficult to define. Consequently, some studies in opossums, rats, cats and monkeys have reported the endopiriform nucleus as a portion of the ventral nucleus of the claustrum (Olson and Graybiel, 1980; Witter et al., 1988; Kowiański et al., 1999a; Neville and Haberly, 2004), while others considered the endopiriform nucleus a nucleus independent from it (Valverde, 1965; Beneyto and Prieto, 2001).

To our knowledge, this the first study reporting the quantification of different subtypes of GABAergic neurons in the PC and endopiriform nucleus of the ferret. The immunolabeling features (cell shape, and bouton distribution) of GAD67-ir, CR-ir and PV-ir neurons was similar to that described in the PC of rodents (Celio, 1990; Frassoni et al., 1998; Ekstrand et al., 2001a) and opossums (Haberly et al., 1987). However, the morphology of GABAergic neurons is highly variable and the profusion of forms may actually reflect different functional roles in the cerebral cortex (Ekstrand et al., 2001a; DeFelipe et al., 2013; Taniguchi, 2014). In summary, five types of GABAergic neurons have been reported in the PC of several vertebrates, principally rats and opossums (Haberly et al., 1987; Westenbroek et al., 1987; Kubota and Jones, 1993; Frassoni et al., 1998; Ekstrand et al., 2001a; Neville and Haberly, 2004). These neuronal types are the bipolar or bi-tufted cells, which are a source of perisomatic CR-ir endings on pyramidal cells, and the large horizontal and the small multipolar cells located in layer I, which establish synapses on dendrites of pyramidal neurons (Sanides-Kohlrausch and Wahle, 1990; Ekstrand et al., 2001a; Larriva-Sahd, 2010). The remaining two types are the basket and chandelier cells, which are the major source of perisomatic basket and axon initial segment inhibitory endings and both, are PV-ir (DeFelipe et al., 1989). Basket cells are large multipolar cells, principally located in layer II and superficial layer III, and their axons can give rise to long branches, parallel to the pial surface, providing a potential substrate for lateral inhibition (Ekstrand et al., 2001b). Chandelier cells have several long apical dendrites and usually a basal axon that establishes synapses onto the initial axon segments of pyramidal cells in PC layers II–III (Somogyi et al., 1982, 1983; Haberly and Presto, 1986; Ekstrand et al., 2001b; Larriva-Sahd, 2010). The PV-ir chandelier-like axon terminals, abundant in the primate neocortex (e.g., DeFelipe et al., 1989), were scarcer in our study in the piriform network of the ferret. Decreased perisomatic and axon initial segment inhibition might have important functional consequences such as loss of control of the pattern and timing of pyramidal neuron output, resulting in a loss of synchronization of their response (Freund, 2003; Markram et al., 2004; Klausberger and Somogyi, 2008). The above mentioned five neuronal types have been categorized in the neocortex of mice into PV-ir (which include fast-spiking basket and chandelier cells, and accounts for approximately 40% of all GABAergic neurons), somatostatin-ir (which includes CR-ir cells) and 5′HT3aR expressing cells (Rudy et al., 2011). In any case, we aimed to quantify the distribution of the principal types of GABAergic neurons of the piriform network that might have a prominent role in epilepsy, rather than to describe all GABAergic neuron types.

### Cytoarchitecture of Visual Areas 17, 18 and 19

Overall, the distribution in all visual areas of GAD67-ir and PV-ir neurons was spread across layers II–VI, whereas CR-ir neurons were densest in layers II–III, progressively less dense in layers IV–VI, and layer I contained the lowest number of cells (Figures 2, 3).

Our results indicate that in visual areas, 28.8% neurons were GAD67-ir, 14.8% were CR-ir and 15.8% were PV-ir (Figures 7E–G; Supplementary Table S1). We have quantified the major types of GABAergic neurons in the ferret visual areas, but our estimations remain an underestimation of the total number of GABAergic neurons. For instance, GABAergic neurons expressing GAD65, 5′HT3aR receptors (excluding CR-ir) and somatostatin (Rudy et al., 2011) were not quantified. Nonetheless, our data are consistent with other studies reporting that approximately 20% of neurons are GABAergic in the mouse neocortex (Sahara et al., 2012) and around 25%–30% in the primate neocortex (Jones, 1993; reviewed by Kubota, 2014). The most relevant GABAergic neurons are PV-ir and comprise basket (synapsing on the soma) and chandelier neurons (synapsing on axon initial segments). PV-ir neurons are crucial in controlling the excitatory output of pyramidal neurons (Somogyi et al., 1982) and play a key role in epilepsy (DeFelipe, 1999). In the rodent somatosensory cortex, PV-ir neurons account for about 40% of total GABAergic interneurons (Uematsu et al., 2008; Xu et al., 2010), strongly inhibit neighboring excitatory pyramidal neurons and are often fast-spiking, characterized by a high frequency train of action potentials with little adaptation (Kawaguchi et al., 1987; Cauli et al., 1997; Kawaguchi, 1997; Xu and Callaway, 2009). PV-ir basket neurons may be reciprocally connected to neighboring pyramidal neurons and mediate activity-dependent feedforward inhibition (Gabernet et al., 2005). These features may help to generate and maintain cortical network synchronization and oscillation (Tamás et al., 2000; Compte et al., 2008; Taniguchi, 2014). As mentioned above, chandelier neurons form synapses specifically on axon initial segments, which are sites of action potential initiation (Somogyi et al., 1982; Inan et al., 2013). Thus, chandelier neurons fully control spike initiation and thereby synchronize activity of neuronal ensembles (Freund, 2003).

In conclusion, GABAergic neuron proportion was higher in primary visual area 17 compared to secondary areas 18 and 19. In particular, GAD67-ir and PV-ir neuron proportion were higher in area 17 than in secondary areas 18 and 19, while CR-ir neuron proportion was similar in areas 17, 18 and 19. Consistently, VGluT1-ir/VGAT-ir bouton ratio was lower in area 17 (2.5) than in areas 18 and 19 (on average, 2.7).

### The Piriform Network and Visual Areas of the Ferret: Similarities and Differences

The comparison of two cerebral regions that developed at completely different time points during evolution such as the piriform network (paleocortex) and the visual cortex (neocortex) has been controversial. The three-layer PC can be considered a complete network module and from a functional point of view cannot be compared with layers I–III of visual areas that are associative input-output layers, requiring interaction with other layers to do something computationally meaningful. None the less, we have chosen to compare layers I–III of the PC with layers I–III of the visual cortex, and the piriform network with layers I–VI of the visual cortex of these two phylogenetically and functionally different cortical areas because of the physiological evidence pointing towards a higher excitability of the PC with respect to the visual cortex (Piredda and Gale, 1985). The PC is known to be prone to the initiation of seizures (Löscher and Ebert, 1996).

The percentage of CR-ir and PV-ir neurons along layers I–III was similar between piriform and visual cortices, with most CR-ir neurons being equally distributed between layers II and III, and with a progressive increase of PV-ir neurons from layers I to III (Figures 7A–C). The other similarity was that the density and size of VGluT1-ir and VGAT-ir boutons was similar across layers I–III, although the density and size of VGAT-ir boutons were slightly higher in the visual cortex (see Figures 10A,B,E,F). Despite these similarities, the piriform network had 49.8% fewer GAD67-ir neurons, 25.5% fewer CR-ir neurons and 58.2% fewer PV-ir neurons than the visual cortex (Figures 7D–G). Although the proportion of PV-ir neurons in the piriform network was significantly lower than in the visual areas, the percentage of PV-ir neurons was significantly higher in layer I of the PC compared with the visual areas (15.8% vs. 1.1%). The function of PV-ir neurons in layer I of the ferret PC remains unknown. Unlike neocortical areas, layer I of the PC is an important target of sensory inputs. For instance, layer Ia receives afferents from the olfactory bulb and layer Ib from the anterior olfactory nucleus and the entorhinal cortex (Powell et al., 1965; White, 1965; Luskin and Price, 1983a; Manger et al., 2002a; Haberly, 1990). Therefore, one of the key roles of layer I PV-ir neurons in the ferret PC might be the regulation of these inputs, affecting the generation of action potentials of the superficial layer II pyramidal neurons. Also different from the PC is the organization of the neocortex, which is assembled in functional cortical columns where the specific distribution of GABAergic neurons plays an important role (Markram et al., 2004). In the PC, although it has a non-columnar organization (Johnson et al., 2000), the different distribution and number of PV-ir neurons in layers II–III, compared with visual areas, suggests that not only the lateral inhibition but also the synchronization of firing of principal neurons, differs from visual areas.

In addition, to a lower proportion of inhibitory cells, the piriform network also shows a higher VGluT1-ir/VGAT-ir bouton ratio than the visual cortex and these ratios along layers I–III also differed between the two regions (Figures 15C,D,G,H). In particular, the piriform network presented over 3.1 times more glutamatergic than GABAergic boutons, higher than the 2.7 ratio observed in the visual cortex of ferrets and the 2.4 ratio in layers I–III of the somatosensory cortex of rats (Navarro et al., 2015).

The inhibition levels of the piriform network are not solely determined by the density of GABAergic neurons and of glutamatergic and GABAergic boutons, but also by properties of the system such as the firing patterns, the functional strength of the synapses and the diversity of other types of neurons and glial cells. However, the lower proportion of GABAergic neurons (fundamentally PV-ir neurons) and the higher VGluT1-ir/VGAT-ir bouton ratio in the piriform network in comparison to visual areas might be an important cytoarchitectonic substrate affecting the excitability and speed of slow wave propagation found in the piriform network (Sanchez-Vives et al., 2008, 2010) and might therefore have important functional consequences, resulting in the tendency of this area to initiate epileptic discharges (Piredda and Gale, 1985). Since the piriform network presents this excitatory/inhibitory profile, our data may provide important cues regarding its susceptibility to epileptic activity.

## Author Contributions

The conception, design and draft of the work were carried out by PP, MS-V and PB. The acquisition, analysis and interpretation of histological data were performed by DN, MA, AF, CG-L, FS-L and PB. All authors contributed to discussing the results and writing specific parts of the manuscript, and all approved the final version of the manuscript.
