# Supplementary material for: Distribution of GABAergic Neurons and VGluT1 and VGAT Immunoreactive Boutons in the Ferret (Mustela putorius) Piriform Cortex and Endopiriform Nucleus. Comparison With Visual Areas 17, 18 and 19
Source: Front Neuroanat. 2019 May 31;13:54. doi: 10.3389/fnana.2019.00054 (PMC6554450; doi:10.3389/fnana.2019.00054)
Supplement: Supplementary file 1 [file Data_Sheet_1.zip › Supplementary Figure 1.pdf]

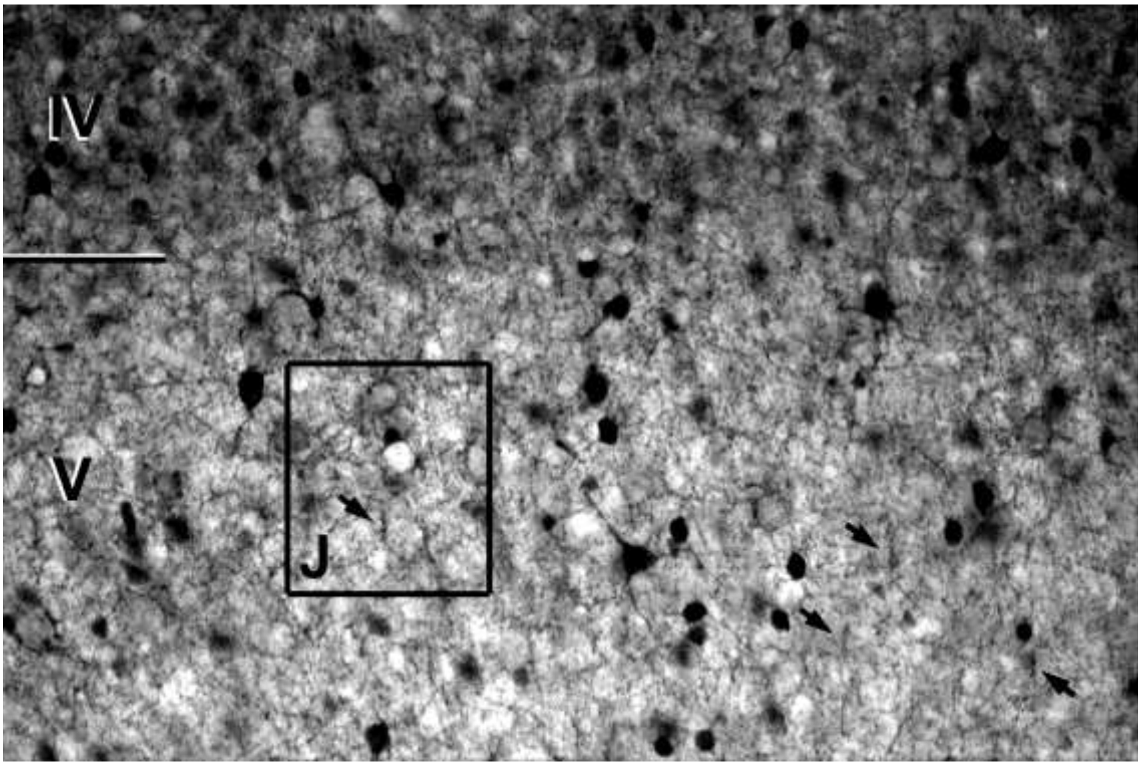

Supplementary Figure S1. Immunostaining of PV-ir neurons in visual area 17. Photomicrograph shows detail of the distribution of PV-ir neurons in layers IV and V of area 17. Note the increased PV immunostaining in layer IV and PV-ir boutons resembling chandelier-like cartridges (arrows). As a reference, the **J** box is indicated in **Figures 5H,I**. Some parent neuronal somata cannot be seen most likely because they are in an adjacent parasagittal section.
