# Supplementary material for: Distribution of GABAergic Neurons and VGluT1 and VGAT Immunoreactive Boutons in the Ferret (Mustela putorius) Piriform Cortex and Endopiriform Nucleus. Comparison With Visual Areas 17, 18 and 19
Source: Front Neuroanat. 2019 May 31;13:54. doi: 10.3389/fnana.2019.00054 (PMC6554450; doi:10.3389/fnana.2019.00054)
Supplement: Supplementary file 1 [file Data_Sheet_1.zip › Supplementary Figure 2.pdf]

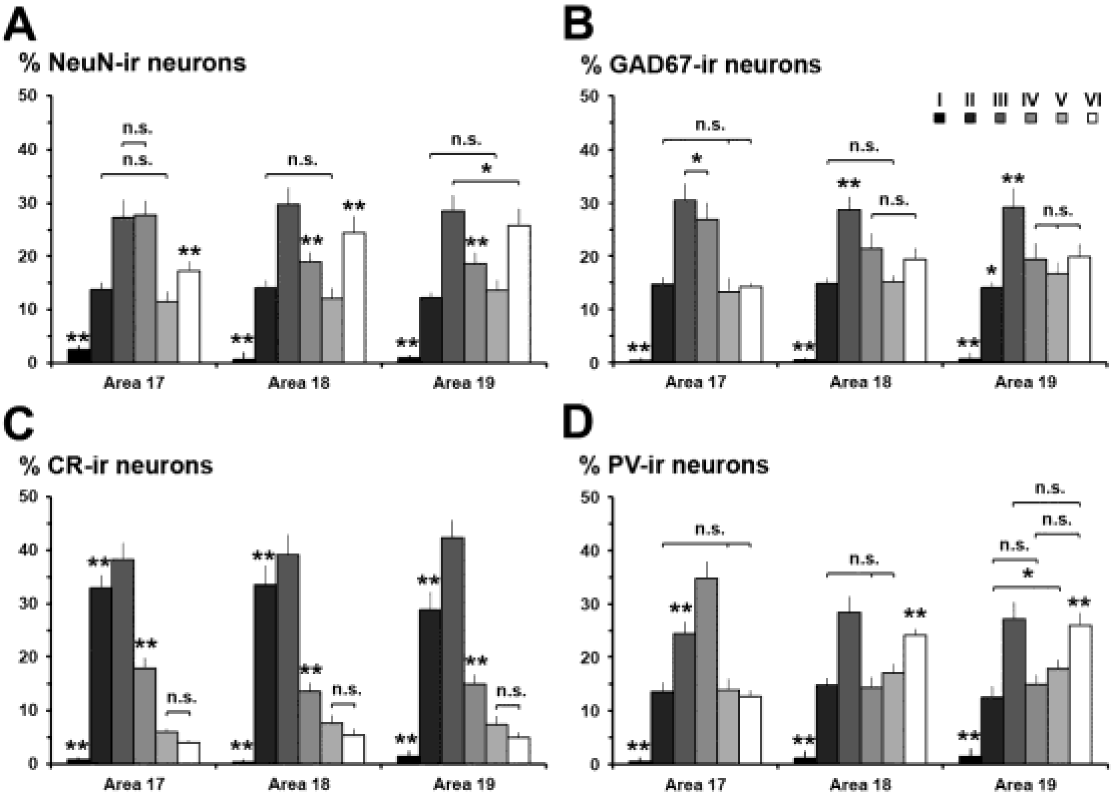

Supplementary Figure S2. Distribution of NeuN-ir, GAD67-ir, CR-ir and PV-ir neurons in visual areas. (**A-D**) Bar charts show the percentages of labelled neurons in cortical layers grouped by visual areas. In all areas, lowest percentages were found in layer I. (**A,B,D**) NeuN-ir, GAD67-ir and PV-ir neuron percentages in layer IV of area 17 was higher than in areas 18 and 19. (**C**) CR-ir neuron percentage distributions were similar among areas. The CR-ir neuron percentage was highest in layers II-III. Vertical lines in bar chart show SD. n.s.: not significant differences. **P* < 0.05, ***P* < 0.001.
