# Supplementary material for: Distribution of GABAergic Neurons and VGluT1 and VGAT Immunoreactive Boutons in the Ferret (Mustela putorius) Piriform Cortex and Endopiriform Nucleus. Comparison With Visual Areas 17, 18 and 19
Source: Front Neuroanat. 2019 May 31;13:54. doi: 10.3389/fnana.2019.00054 (PMC6554450; doi:10.3389/fnana.2019.00054)
Supplement: Supplementary file 1 [file Data_Sheet_1.zip › Supplementary IHC protocols.pdf]

Supplementary Immunohistochemistry (IHC) protocols.

Anti-glutamic acid decarboxylase 67 (GAD67) monoclonal antibody (mAb) protocol

1. Wash in phosphate buffer-saline (PBS; 0.15M NaCl in 0.1M PB). 3*10 min at room temp (rt).
2. Antigen retrieval in DAKO (citrate buffer, pH 6.1; ref. S1699, DAKO North America, Carpinteria, CA, USA). 20 min at 95ºC. See manufacturer datasheet instructions.
3. Wash in PBS-ethanol (PBS and 40% ethanol; 1:1). 3*10 min at rt.
4. Incubate in 30% H_2O_2 in PBS-ethanol (1:9). 30 min rt.
5. Wash in PBS. 3*10 min at rt.
6. Incubate in blocking solution (BS; 3% normal horse serum, 0.1% Triton X-100 in PBS). 1 hr at rt.
7. Incubate in mouse anti-GAD67 mAb in BS (1:2000; Chemicon International Inc., Temecula, CA, USA). 2 d at 4ºC
8. Wash in PBS. 3*10 min at rt.
9. Incubate in horse anti-mouse biotinylated Ab in BS (1:150; Vector Laboratories Inc., Burlingame, CA, USA). 2 hr at rt.
10. Wash in PBS. 3*10 min at rt.
11. Incubate in Vectastain ABC in PBS (1:200; Vector Laboratories Inc.). 1 hr rt.
12. Wash in PBS. 3*10 min at rt.
13. Incubate in 0.02% 3,3'diaminobenzidine (DAB, Sigma-Aldrich Co. St. Louis, MO, USA) in PBS. 5 min at rt.
14. Incubate in 0.05% DAB and 0.009% H_2O_2 in PBS. 10-20 min at rt. Check the intensity of the staining.
15. Wash in PBS. 3*10 min at rt.

Anti-neuronal nuclei (NeuN) and anti-parvalbumin (PV) monoclonal antibodies (mAb) protocols

Note: for the abbreviations definitions and solutions components see the GAD67 protocol.

1. Wash in PBS. 3*10 min at rt.
2. Wash in PBS-ethanol (1:1). 3*10 min at rt.
3. Incubate in 30% H_2O_2 in PBS-ethanol (1:9). 30 min rt.
4. Wash in PBS. 3*10 min at rt.
5. Incubate in BS (3% normal horse serum, 0.1% Triton X-100 in PBS). 1 hr at rt.
6. Incubate in mouse anti-NeuN mAb in BS (1:400; Chemicon International Inc.) or mouse anti- PV mAb (1:1000; Swant, Bellinzona, Switzerland). Overnight at 4ºC.
7. Wash in PBS. 3*10 min at rt.
8. Incubate in horse anti-mouse biotinylated Ab in BS (1:150; Vector Laboratories Inc.). 2 hr at rt.
9. Wash in PBS. 3*10 min at rt.
10. Incubate in Vectastain ABC in PBS (1:200; Vector Laboratories Inc.). 1 hr rt.
11. Wash in PBS. 3*10 min at rt.
12. Incubate in 0.02% DAB (Sigma-Aldrich Co.) in PBS. 5 min at rt.
13. Incubate in 0.05% DAB and 0.009% H_2O_2 in PBS. 10-20 min at rt. Check the intensity of the staining.
14. Wash in PBS. 3*10 min at rt.

Anti-calretinin (CR) protocols

Note: for the abbreviations definitions and solutions components see the GAD67 protocol.

1. Wash in PBS. 3*10 min at rt.
2. Wash in PBS-ethanol (1:1). 3*10 min at rt.
3. Incubate in 30% H_2O_2 in PBS-ethanol (1:9). 30 min rt.
4. Wash in PBS. 3*10 min at rt.
5. Incubate in BS (3% normal goat serum, 0.1% Triton X-100 in PBS). 1 hr at rt.
6. Incubate in rabbit anti-CR Ab in BS (1:2000; Swant). Overnight at 4ºC.
7. Wash in PBS. 3*10 min at rt.
8. Incubate in goat anti-rabbit biotinylated Ab in BS (1:150; Vector Laboratories Inc.). Overnight at 4ºC.
9. Wash in PBS. 3*10 min at rt.
10. Incubate in Vectastain ABC in PBS (1:200; Vector Laboratories Inc.). 1 hr rt.
11. Wash in PBS. 3*10 min at rt.
12. Incubate in 0.02% DAB (Sigma-Aldrich Co.) in PBS. 5 min at rt.
13. Incubate in 0.05% DAB and 0.009% H_2O_2 in PBS. 10-20 min at rt. Check the intensity of the staining.
14. Wash in PBS. 3*10 min at rt.

Anti-vesicular glutamate transporter 1 (VGluT1) policlonal antibody (Ab) and anti-vesicular GABA transporter (VGAT) Ab protocol

Note: for the abbreviations definitions and solutions components see the GAD67 protocol.

1. Wash in PBS. 3*10 min at rt.
2. Incubate in BS (3% normal goat serum, 0.1% Triton X-100 in PBS). 1 hr at rt.
3. Incubate in rabbit anti-CR Ab in BS (1:2000; Swant). Overnight at 4ºC.
4. Wash in PBS. 3*10 min at rt.
5. Incubate in guinea pig anti-VGluT1 Ab (1:5000; Merck-Millipore, Darmstadt, Germany) and in rabbit anti-VGAT Ab (1:2000; Synaptic Systems, Göttingen, Germany) in BS. Overnight at 4ºC.
6. Wash in PBS. 3*10 min at rt.
7. Incubate in goat anti-guinea pig antibody, Alexa Fluor 488 labelled (1:200, Molecular Probes, Eugene, OR, USA) in PBS. 1 hr at rt.
8. Wash in PBS. 3*10 min at rt.
9. Incubate in goat biotinylated anti-rabbit antibody (1:200, Vector Laboratories Inc.) in PBS. 1 hr at rt.
10. Wash in PBS. 3*10 min at rt.
11. Incubate in NeutrAvidin, Rhodamine Red conjugate (1 µg/mL, Molecular Probes) in PBS. 3 hr at rt.
12. Wash in PBS. 3*10 min at rt.
13. Store in PBS at 4ºC until the study of sections in the confocal microscope.
