# Supplementary material for: Distribution of GABAergic Neurons and VGluT1 and VGAT Immunoreactive Boutons in the Ferret (Mustela putorius) Piriform Cortex and Endopiriform Nucleus. Comparison With Visual Areas 17, 18 and 19
Source: Front Neuroanat. 2019 May 31;13:54. doi: 10.3389/fnana.2019.00054 (PMC6554450; doi:10.3389/fnana.2019.00054)
Supplement: Supplementary file 1 [file Data_Sheet_1.zip › Supplementary Table 1.pdf]

Supplementary Table S1. Sampled area, density and percentage of neurons in the piriform cortex (PC) and endopiriform nucleus (EP).

	Sampled area (mm^2)	Density (neurons/105 μm2) and percentage (in parentheses)			
**Anterior**		**NeuN-ir**	**GAD67-ir**	**CR-ir**	**PV-ir**
**PC layer I**	0.860 ± 0.067	17.0 ± 1.1 (4.6 ± 1.0)	4.8 ± 0.3 (9.2 ± 2.4)	2.3 ± 0.2 (5.7 ± 0.9)	4.6 ± 0.3 (16.5 ± 1.8)
**PC layer II**	0.563 ± 0.050	265.3 ± 13.3 (47.5 ± 4.3)	13.4 ± 0.7 (16.8 ± 1.7)	20.8 ± 1.0 (34.5 ± 3.4)	10.4 ± 0.5 (24.2 ± 5.5)
**PC layer III**	1.158 ± 0.093	74.7 ± 7.0 (27.5 ± 3.4)	21.3 ± 2.0 (54.9 ± 4.0)	10.8 ± 1.0 (36.8 ± 4.9)	10.1 ± 0.9 (48.4 ± 6.4)
**PC all layers**	2.582 ± 0.124	97.1 ± 12.1 (79.7 ± 5.9)	14.1 ± 1.8 (80.9 ± 7.1)	10.1 ± 1.3 (77.0 ± 9.6)	8.3 ± 1.0 (89.1 ± 9.1)
**EP**	0.367 ± 0.018	174.1 ± 3.2 (20.3 ± 2.4)	23.4 ± 0.4 (19.1 ± 2.5)	21.3 ± 0.4 (23.0 ± 5.0)	7.2 ± 0.1 (10.9 ± 2.3)
**PC+EP**	2.949 ± 0.117	106.6 ± 12.5 (100)	15.2 ± 1.8 (100)	11.5 ± 1.3 (100)	8.2 ± 1.0 (100)
**Posterior**					
**PC layer I**	1.315 ± 0.210	6.4 ± 0.5 (1.9 ± 0.6)	4.8 ± 0.1 (9.9 ± 0.8)	1.9 ± 0.0 (5.4 ± 1.8)	3.0 ± 0.1 (15.0 ± 2.0)
**PC layer II**	0.783 ± 0.066	218.4 ± 10.3 (39.5 ± 4.2)	13.5 ± 0.9 (16.5 ± 1.6)	20.5 ± 1.9 (34.4 ± 4.1)	7.2 ± 0.7 (21.5 ± 2.0)
**PC layer III**	1.615 ± 0.255	92.3 ± 9.6 (34.4 ± 4.5)	22.1 ± 3.6 (55.8 ± 3.6)	11.3 ± 1.0 (39.1 ± 3.0)	7.5 ± 0.9 (46.2 ± 5.6)
**PC all layers**	3.712 ± 0.311	88.5 ± 11.5 (75.8 ± 9.9)	14.1 ± 2.5 (82.2 ± 4.5)	9.9 ± 1.7 (78.9 ± 6.4)	5.8 ± 0.8 (82.6 ± 6.5)
**EP**	0.367 ± 0.018	285.2 ± 9.2 (24.2 ± 4.4)	30.9 ± 1.6 (17.8 ± 1.8)	26.7 ± 2.5 (21.1 ± 2.9)	12.4 ± 0.2 (17.4 ± 4.7)
**PC+EP**	4.079 ± 0.311	106.1 ± 9.9 (100)	15.7 ± 2.9 (100)	11.4 ± 2.2 (100)	6.4 ± 1.0 (100)

Data are mean ± SD.
