# Supplementary material for: Distribution of GABAergic Neurons and VGluT1 and VGAT Immunoreactive Boutons in the Ferret (Mustela putorius) Piriform Cortex and Endopiriform Nucleus. Comparison With Visual Areas 17, 18 and 19
Source: Front Neuroanat. 2019 May 31;13:54. doi: 10.3389/fnana.2019.00054 (PMC6554450; doi:10.3389/fnana.2019.00054)
Supplement: Supplementary file 1 [file Data_Sheet_1.zip › Supplementary Table 2.pdf]

Supplementary Table S2. Sampled area, density and percentage of neurons in visual areas 17, 18 and 19.

	Sampled area (mm^2)	Density (neurons/10^5 μm^2) and percentage (in parentheses)			
**Area 17**		**NeuN-ir**	**GAD67-ir**	**CR-ir**	**PV-ir**
**I**	0.097 ± 0.004	37.4 ± 14.2 (2.5 ± 0.9)	2.2 ± 0.8 (0.5 ± 0.1)	2.0 ± 0.6 (0.9 ± 0.4)	1.7 ± 0.6 (0.6 ± 0.6)
**II**	0.116 ± 0.005	173.1 ± 40.0 (13.7 ± 1.3)	57.7 ± 18.5 (14.7 ± 1.2)	61.5 ± 19.0 (32.9 ± 2.3)	30.7 ± 5.6 (13.6 ± 1.6)
**III**	0.319 ± 0.013	125.3 ± 28.8 (27.3 ± 3.3)	43.5 ± 10.2 (30.4 ± 3.3)	26.0 ± 8.0 (38.2 ± 3.0)	20.0 ± 4.5 (24.4 ± 2.3)
**IV**	0.235 ± 0.010	173.0 ± 40.1 (27.7 ± 2.8)	52.1 ± 15.5 (26.8 ± 3.2)	16.6 ± 4.6 (17.9 ± 1.9)	38.7 ± 8.7 (37.4 ± 3.1)
**V**	0.140 ± 0.006	120.2 ± 31.4 (11.5 ± 1.9)	43.5 ± 16.5 (13.4 ± 2.4)	9.3 ± 2.1 (6.0 ± 0.6)	26.1 ± 7.1 (14.0 ± 2.0)
**VI**	0.134 ± 0.006	189.6 ± 34.6 (17.3 ± 1.7)	48.7 ± 18.5 (14.3 ± 0.6)	6.6 ± 1.0 (4.0 ± 0.4)	28.4 ± 6.4 (12.7 ± 1.2)
**Area 18**					
**I**	0.149 ± 0.014	8.1 ± 1.4 (0.8 ± 0.1)	1.8 ± 0.3 (0.6 ± 0.1)	0.7 ± 0.1 (0.4 ± 0.4)	1.8 ± 0.3 (1.2 ± 0.1)
**II**	0.122 ± 0.011	177.7 ± 30.3 (14.1 ± 0.9)	55.2 ± 9.4 (14.9 ± 0.9)	61.3 ± 10.4 (33.5 ± 3.5)	27.7 ± 4.7 (14.9 ± 0.8)
**III**	0.418 ± 0.039	109.0 ± 18.6 (29.7 ± 1.2)	31.0 ± 5.3 (28.7 ± 2.4)	20.8 ± 3.5 (39.2 ± 3.7)	15.4 ± 2.6 (28.4 ± 1.9)
**IV**	0.208 ± 0.019	139.3 ± 23.7 (18.9 ± 1.8)	46.2 ± 7.9 (19.2 ± 2.9)	16.4 ± 2.5 (13.7 ± 1.5)	15.7 ± 2.7 (14.4 ± 0.8)
**V**	0.173 ± 0.016	107.2 ± 18.2 (12.1 ± 1.1)	39.4 ± 6.7 (17.2 ± 1.2)	9.9 ± 1.7 (7.7 ± 1.5)	22.3 ± 3.8 (17.1 ± 1.1)
**VI**	0.219 ± 0.020	170.7 ± 29.1 (24.4 ± 2.0)	40.0 ± 6.8 (19.4 ± 2.0)	5.6 ± 0.9 (5.5 ± 1.0)	24.9 ± 4.2 (24.0 ± 0.9)
**Area 19**					
**I**	0.225 ± 0.016	6.7 ± 1.1 (1.1 ± 0.4)	1.0 ± 0.2 (0.7 ± 0.2)	1.2 ± 0.2 (1.4 ± 1.1)	1.3 ± 0.2 (1.5 ± 1.4)
**II**	0.168 ± 0.012	101.4 ± 17.3 (12.3 ± 0.9)	28.6 ± 5.2 (14.1 ± 0.9)	33.6 ± 5.9 (33.8 ± 3.1)	14.5 ± 2.6 (12.5 ± 2.1)
**III**	0.503 ± 0.035	78.5 ± 13.4 (28.5 ± 2.9)	19.8 ± 3.6 (29.2 ± 3.5)	16.5 ± 2.9 (37.1 ± 3.2)	10.5 ± 1.9 (27.2 ± 3.1)
**IV**	0.184 ± 0.013	140.0 ± 23.8 (18.7 ± 1.9)	36.0 ± 6.5 (19.5 ± 2.9)	15.8 ± 2.8 (15.0 ± 1.7)	15.9 ± 2.3 (15.1 ± 1.7)
**V**	0.117 ± 0.008	160.5 ± 27.3 (13.6 ± 1.9)	48.4 ± 8.7 (16.6 ± 2.1)	12.3 ± 2.2 (7.4 ± 1.6)	29.6 ± 5.2 (17.9 ± 1.7)
**VI**	0.257 ± 0.018	138.7 ± 23.6 (25.8 ± 3.1)	26.3 ± 4.8 (19.9 ± 2.3)	3.8 ± 0.7 (5.0 ± 1.0)	19.5 ± 3.4 (25.9 ± 2.4)

Data are mean ± SD.
