# Supplementary material for: Distribution of GABAergic Neurons and VGluT1 and VGAT Immunoreactive Boutons in the Ferret (Mustela putorius) Piriform Cortex and Endopiriform Nucleus. Comparison With Visual Areas 17, 18 and 19
Source: Front Neuroanat. 2019 May 31;13:54. doi: 10.3389/fnana.2019.00054 (PMC6554450; doi:10.3389/fnana.2019.00054)
Supplement: Supplementary file 1 [file Data_Sheet_1.zip › Supplementary Table 3.pdf]

Supplementary Table S3. Sampled area, and density, percentage, proportion, ratio, area and circularity of VGluT1-ir and VGAT-ir boutons in the piriform cortex (PC) and endopiriform nucleus (EP).

	VGluT1-ir boutons			VGAT-ir boutons		
	**Anterior**	**Posterior**	**Total**	**Anterior**	**Posterior**	**Total**
**Sampled area (mm^2)**						
**PC layer I**				1.39 ± 0.064	1.86 ± 0.073	1.62 ± 0.066
**PC layer II**				0.87 ± 0.035	1.22 ± 0.052	1.04 ± 0.042
**PC layer III**				1.77 ± 0.038	2.50 ± 0.074	2.14 ± 0.087
**EP**				0.63 ± 0.018	1.11 ± 0.027	0.87 ± 0.035
**PC+EP**				4.65 ± 0.137	6.69 ± 0.276	5.67 ± 0.230
**Density (boutons/10^3 µm^2)**						
**PC layer I**	282.6 ± 41.4	266.2 ± 31.1	273.2 ± 35.7	85.0 ± 11.7	88.1 ± 8.5	86.8 ± 9.8
**PC layer II**	233.3 ± 33.7	242.2 ± 25.5	238.5 ± 29.3	74.9 ± 8.9	78.7 ± 7.1	77.2 ± 8.0
**PC layer III**	213.0 ± 45.7	242.7 ± 20.0	230.4 ± 37.7	76.3 ± 17.4	75.7 ± 5.4	75.9 ± 11.8
**EP**	270.9 ± 20.8	271.3 ± 58.1	271.1 ± 47.0	80.8 ± 9.5	80.8 ± 4.9	80.8 ± 6.7
**PC+EP**	256.9 ± 30.5	255.6 ± 33.7	250.4 ± 37.7	79.8 ± 11.1	80.8 ± 6.5	80.0 ± 10.2
**Bouton percentage**						
**PC layer I**	34.4 ± 4.0	29.1 ± 1.9	31.8 ± 3.0	32.0 ± 4.0	30.4 ± 1.9	31.2 ± 3.0
**PC layer II**	17.7 ± 3.5	17.4 ± 1.8	17.5 ± 2.6	17.6 ± 3.5	17.8 ± 1.8	17.7 ± 2.6
**PC layer III**	33.1 ± 2.9	35.8 ± 1.9	34.4 ± 2.4	36.7 ± 2.9	35.2 ± 1.9	35.9 ± 2.4
**EP**	14.8 ± 2.1	17.7 ± 3.3	16.3 ± 2.7	13.7 ± 2.1	16.6 ± 3.3	15.2 ± 2.7
**PC+EP**	100	100	100	100	100	100
**Bouton proportion (%) in each layer (VGluT1-ir/VGAT-ir bouton ratio)**						
**PC layer I**				23.4 ± 4.0<br>(3.4)	25.0 ± 1.9<br>(3.0)	24.3 ± 3.0<br>(3.2)
**PC layer II**				24.5 ± 3.5<br>(3.2)	24.6 ± 1.8<br>(3.1)	24.6 ± 2.6<br>(3.1)
**PC layer III**				26.5 ± 2.9<br>(2.8)	23.8 ± 1.9<br>(3.2)	25.2 ± 3.0<br>(3.0)
**EP**				23.0 ± 2.1<br>(3.4)	23.4 ± 3.3<br>(3.4)	23.2 ± 2.8<br>(3.4)
**PC+EP**				23.9 ± 2.8<br>(3.3)	24.2 ± 2.2<br>(3.2)	24.4 ± 2.7<br>(3.2)
**Bouton area (µm^2) and circularity (between parenthesis)**						
**PC layer I**	0.214 ± 0.036<br>(0.88 ± 0.02)	0.213 ± 0.021<br>(0.91 ± 0.01)	0.213 ± 0.027<br>(0.90 ± 0.02)	0.264 ± 0.045<br>(0.64 ± 0.04)	0.259 ± 0.024<br>(0.66 ± 0.02)	0.261 ± 0.033<br>(0.65 ± 0.03)
**PC layer II**	0.214 ± 0.029<br>(0.89 ± 0.01)	0.210 ± 0.018<br>(0.88 ± 0.01)	0.212 ± 0.023<br>(0.89 ± 0.02)	0.311 ± 0.026<br>(0.62 ± 0.02)	0.295 ± 0.031<br>(0.63 ± 0.02)	0.302 ± 0.029<br>(0.63 ± 0.02)
**PC layer III**	0.213 ± 0.015<br>(0.90 ± 0.01)	0.188 ± 0.016<br>(0.90 ± 0.01)	0.201 ± 0.023<br>(0.90 ± 0.02)	0.301 ± 0.051<br>(0.61 ± 0.05)	0.303 ± 0.025<br>(0.65 ± 0.01)	0.302 ± 0.039<br>(0.63 ± 0.03)
**EP**	0.189 ± 0.021<br>(0.91 ± 0.01)	0.174 ± 0.028<br>(0.91 ± 0.01)	0.180 ± 0.026<br>(0.91 ± 0.02)	0.278 ± 0.038<br>(0.63 ± 0.02)	0.261 ± 0.025<br>(0.63 ± 0.01)	0.267 ± 0.031<br>(0.63 ± 0.03)
**PC+EP**	0.201 ± 0.024<br>(0.89 ± 0.01)	0.196 ± 0.021<br>(0.09 ± 0.01)	0.203 ± 0.025<br>(0.89 ± 0.02)	0.285 ± 0.039<br>(0.62 ± 0.04)	0.280 ± 0.026<br>(0.65 ± 0.02)	0.285 ± 0.036<br>(0.64 ± 0.04)

Data are mean ± SD.
