# Supplementary material for: Distribution of GABAergic Neurons and VGluT1 and VGAT Immunoreactive Boutons in the Ferret (Mustela putorius) Piriform Cortex and Endopiriform Nucleus. Comparison With Visual Areas 17, 18 and 19
Source: Front Neuroanat. 2019 May 31;13:54. doi: 10.3389/fnana.2019.00054 (PMC6554450; doi:10.3389/fnana.2019.00054)
Supplement: Supplementary file 1 [file Data_Sheet_1.zip › Supplementary Table 4.pdf]

Supplementary Table S4. Sampled area, and density, percentage, proportion, ratio, area and circularity of VGluT1-ir and VGAT-ir boutons in visual areas 17, 18 and 19.

	**VGluT1-ir boutons**			**VGAT-ir boutons**		
	**Area 17**	**Area 18**	**Area 19**	**Area 17**	**Area 18**	**Area 19**
**Sampled area (x10^5 μm^2)**						
**I**				1.03 ± 0.021	1.57 ± 0.032	2.37 ± 0.049
**II**				1.24 ± 0.026	1.28 ± 0.026	1.77 ± 0.037
**III**				3.41 ± 0.070	4.39 ± 0.061	5.30 ± 0.079
**IV**				2.50 ± 0.052	2.19 ± 0.045	1.95 ± 0.040
**V**				1.50 ± 0.031	1.82 ± 0.038	1.24 ± 0.025
**VI**				1.43 ± 0.029	2.30 ± 0.047	2.72 ± 0.056
**Density (boutons/10^3 μm^2)**						
**I**	291.8 ± 38.7	290.9 ± 16.7	327.6 ± 32.1	121.7 ± 7.3	112.6 ± 5.8	135.8 ± 10.3
**II**	274.8 ± 32.4	275.8 ± 32.7	265.0 ± 13.1	111.8 ± 4.6	100.6 ± 5.0	101.8 ± 4.5
**III**	261.6 ± 35.4	250.2 ± 27.9	258.6 ± 22.4	103.3 ± 9.9	91.0 ± 5.2	94.0 ± 4.2
**IV**	254.9 ± 32.1	243.7 ± 23.1	231.4 ± 10.7	96.1 ± 9.6	83.9 ± 6.4	79.6 ± 6.3
**V**	212.5 ± 43.9	211.5 ± 15.9	211.3 ± 22.1	87.7 ± 4.4	79.1 ± 4.3	78.2 ± 7.2
**VI**	228.8 ± 27.6	220.5 ± 14.4	209.2 ± 25.2	95.1 ± 4.8	84.0 ± 4.8	75.1 ± 11.0
**Bouton percentage**						
**I**	10.7 ± 0.9	13.8 ± 0.4	19.9 ± 1.3	11.2 ± 0.4	14.4 ± 0.3	22.1 ± 1.1
**II**	12.1 ± 1.4	10.0 ± 2.0	12.1 ± 0.8	12.4 ± 0.5	10.5 ± 0.8	12.4 ± 0.7
**III**	31.7 ± 3.8	33.2 ± 4.4	35.2 ± 3.4	31.3 ± 2.7	32.6 ± 2.1	34.2 ± 1.6
**IV**	22.7 ± 3.3	16.1 ± 1.6	11.6 ± 0.8	21.4 ± 2.5	15.0 ± 1.1	10.6 ± 1.2
**V**	11.3 ± 2.3	11.6 ± 1.1	6.7 ± 1.8	11.7 ± 0.6	11.8 ± 0.8	6.6 ± 1.4
**VI**	11.6 ± 1.8	15.3 ± 1.5	14.6 ± 3.3	12.1 ± 0.8	15.8 ± 1.2	14.0 ± 3.7
**Bouton proportion (%) in each layer (VGluT1-ir/VGAT-ir bouton ratio)**						
**I**				29.4 ± 2.2 (2.4)	27.9 ± 1.2 (2.6)	29.3 ± 1.6 (2.4)
**II**				28.9 ± 2.4 (2.5)	28.1 ± 2.6 (2.6)	27.8 ± 1.0 (2.6)
**III**				28.3 ± 3.9 (2.5)	26.7 ± 2.8 (2.7)	26.7 ± 1.4 (2.8)
**IV**				27.4 ± 3.4 (2.7)	25.6 ± 1.7 (2.9)	25.6 ± 1.1 (2.9)
**V**				29.2 ± 4.3 (2.4)	27.2 ± 1.7 (2.7)	27.0 ± 0.8 (2.7)
**VI**				29.4 ± 2.3 (2.4)	27.6 ± 0.8 (2.6)	26.4 ± 3.5 (2.8)
**Bouton area (μm^2) and circularity (between parenthesis)**						
**I**	0.219 ± 0.030 (0.90 ± 0.01)	0.215 ± 0.017 (0.91 ± 0.01)	0.186 ± 0.014 (0.90 ± 0.01)	0.270 ± 0.013 (0.67 ± 0.04)	0.267 ± 0.015 (0.69 ± 0.01)	0.252 ± 0.009 (0.66 ± 0.01)
**II**	0.224 ± 0.042 (0.90 ± 0.01)	0.217 ± 0.019 (0.90 ± 0.01)	0.202 ± 0.013 (0.89 ± 0.01)	0.348 ± 0.033 (0.66 ± 0.02)	0.335 ± 0.015 (0.66 ± 0.02)	0.319 ± 0.009 (0.63 ± 0.01)
**III**	0.204 ± 0.028 (0.89 ± 0.01)	0.225 ± 0.022 (0.90 ± 0.01)	0.219 ± 0.010 (0.90 ± 0.01)	0.361± 0.028 (0.66 ± 0.02)	0.366 ± 0.021 (0.65 ± 0.02)	0.339 ± 0.029 (0.62 ± 0.01)
**IV**	0.219 ± 0.029 (0.89 ± 0.01)	0.219 ± 0.025 (0.89 ± 0.01)	0.218 ± 0.049 (0.88 ± 0.01)	0.367 ± 0.022 (0.66 ± 0.02)	0.384 ± 0.020 (0.61 ± 0.03)	0.356 ± 0.059 (0.58 ± 0.05)
**V**	0.245 ± 0.033 (0.90 ± 0.01)	0.219 ± 0.024 (0.89 ± 0.01)	0.212 ± 0.047 (0.88 ± 0.02)	0.357 ± 0.017 (0.66 ± 0.02)	0.359 ± 0.023 (0.60 ± 0.05)	0.378 ± 0.052 (0.56 ± 0.07)
**VI**	0.258 ± 0.027 (0.89 ± 0.01)	0.234 ± 0.016 (0.89 ± 0.01)	0.247 ± 0.012 (0.88 ± 0.02)	0.361 ± 0.031 (0.64 ± 0.02)	0.337 ± 0.010 (0.60 ± 0.05)	0.359 ± 0.051 (0.56 ± 0.05)

Data are mean ± SD.
